# Supplementary material for: Kisspeptin improves local ovarian insulin resistance in PCOS by modulating the PI3K/AKT/GLUT4 signaling pathway
Source: PLoS One. 2026 Feb 2;21(2):e0342158. doi: 10.1371/journal.pone.0342158 (PMC12863573; doi:10.1371/journal.pone.0342158)

**actin**
**Grouping from left to right:** PCOS-IR, Kisspeptin, Kisspeptin 234 TFA

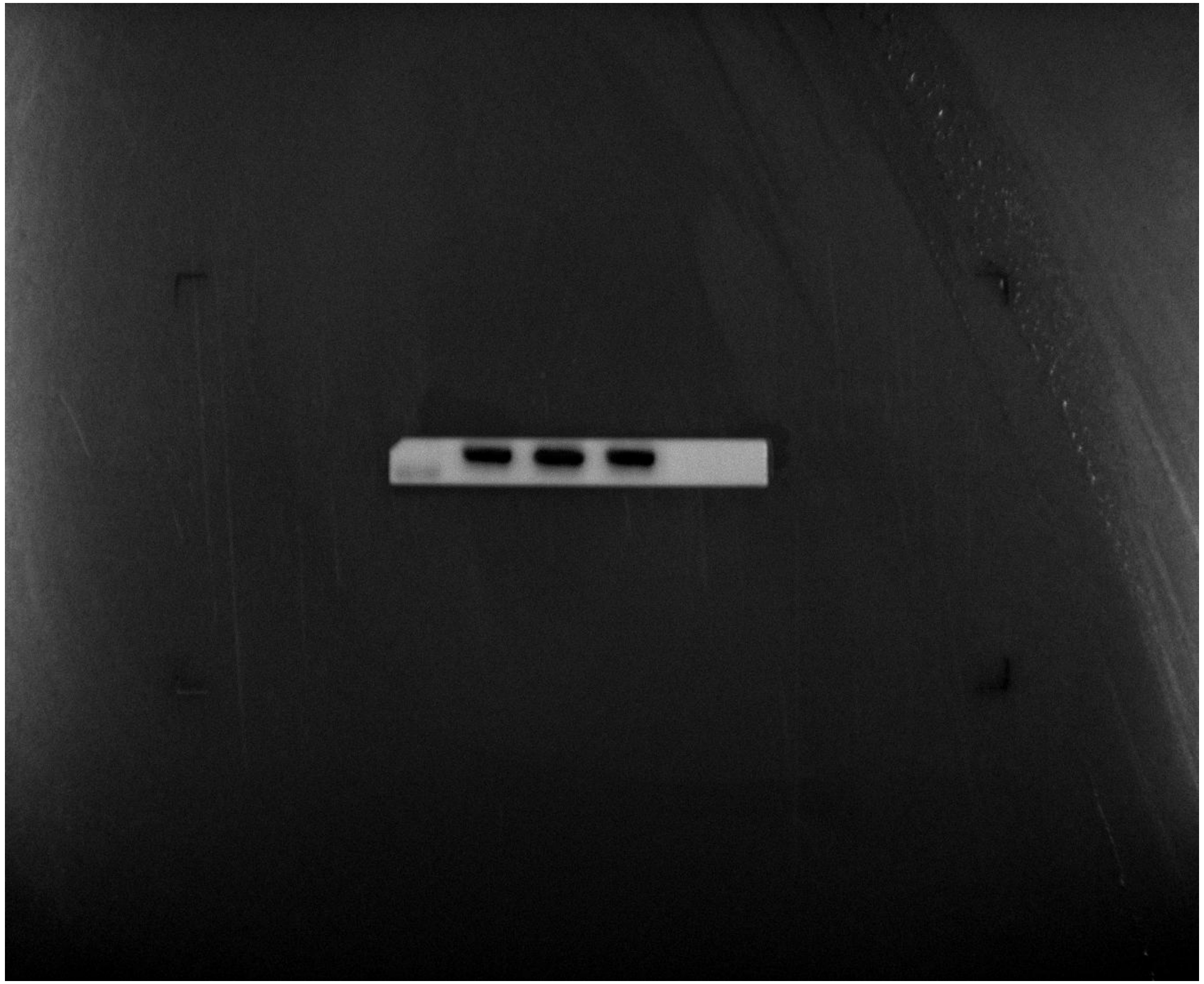

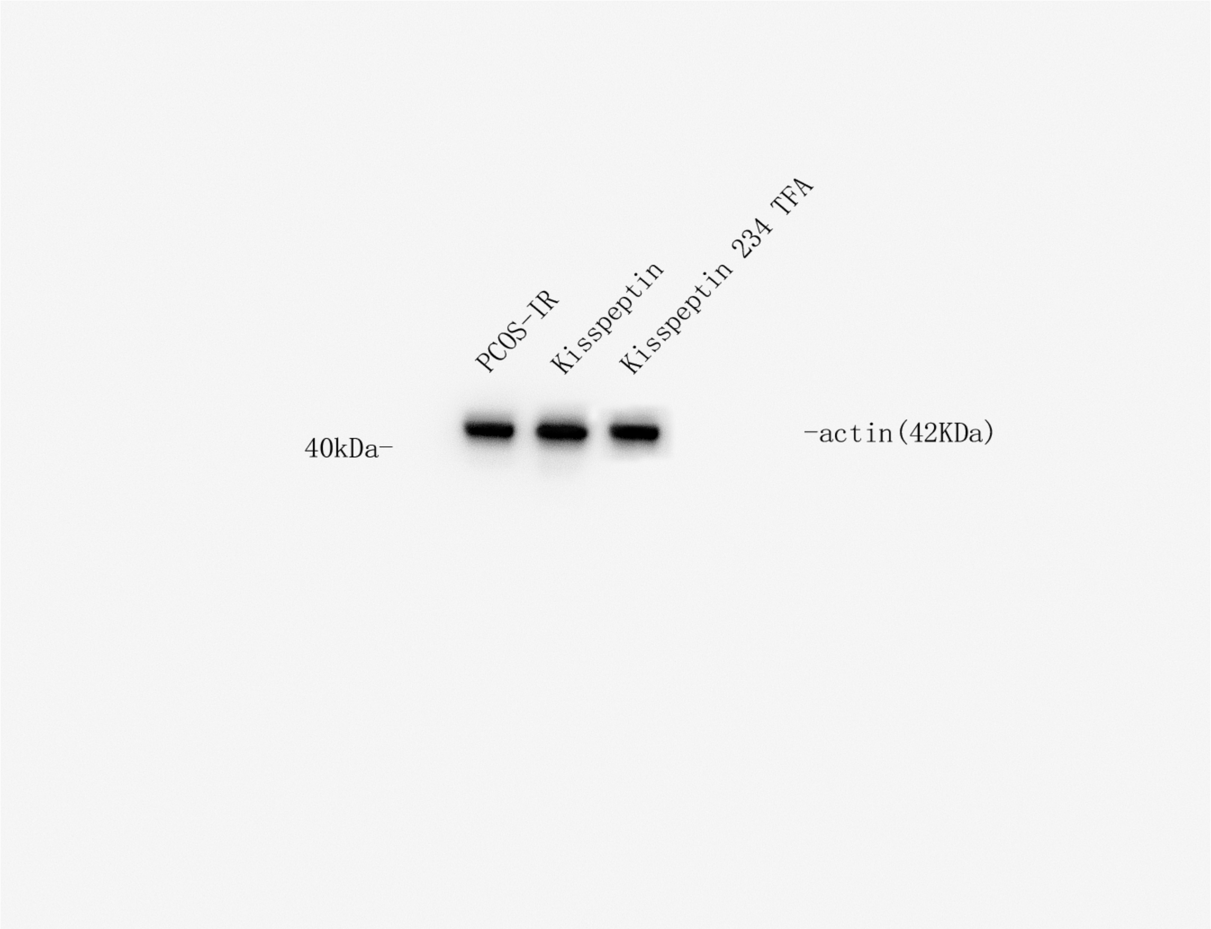

**p-AKT**

**Grouping from left to right:** PCOS-IR, Kisspeptin, Kisspeptin 234 TFA

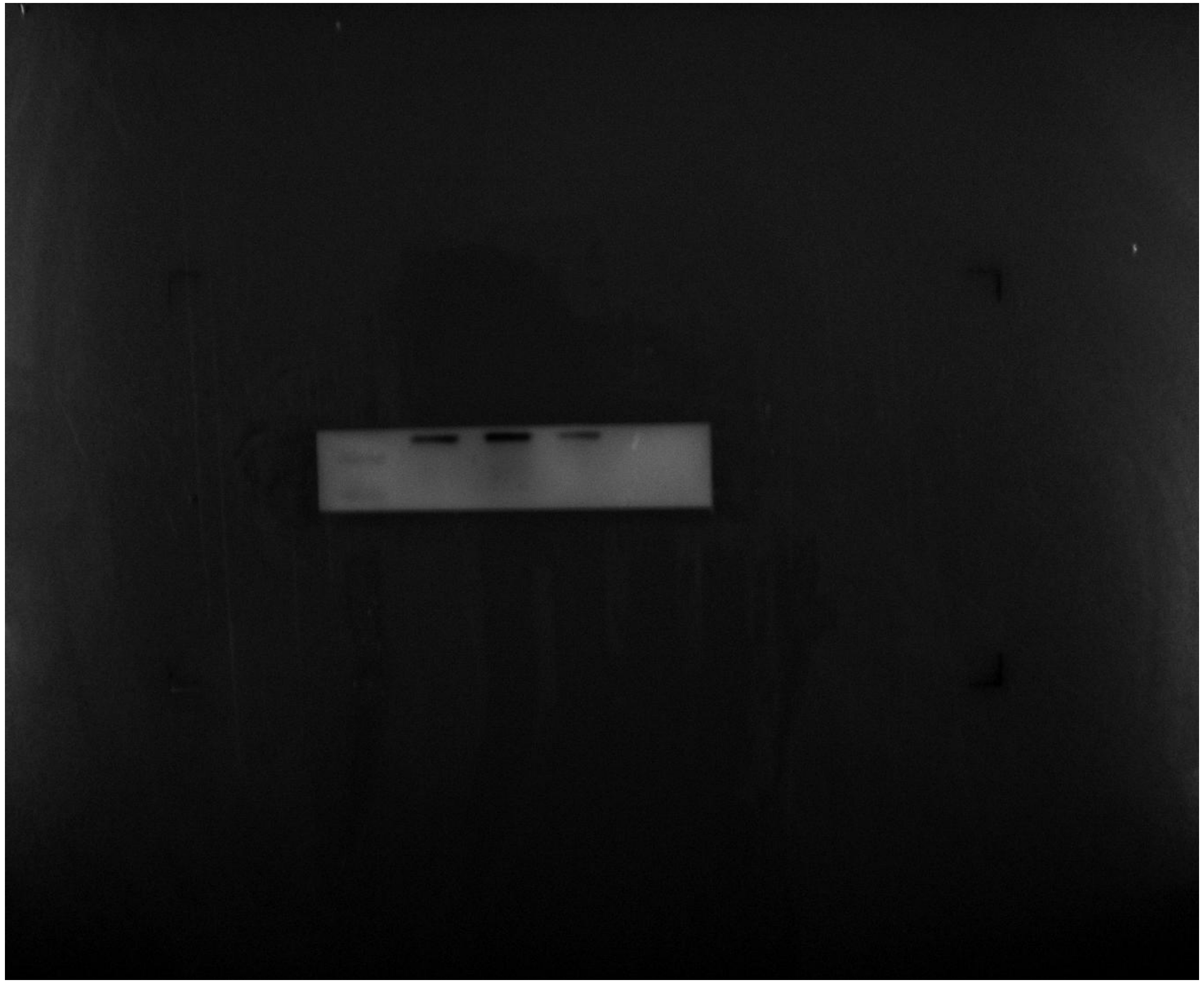

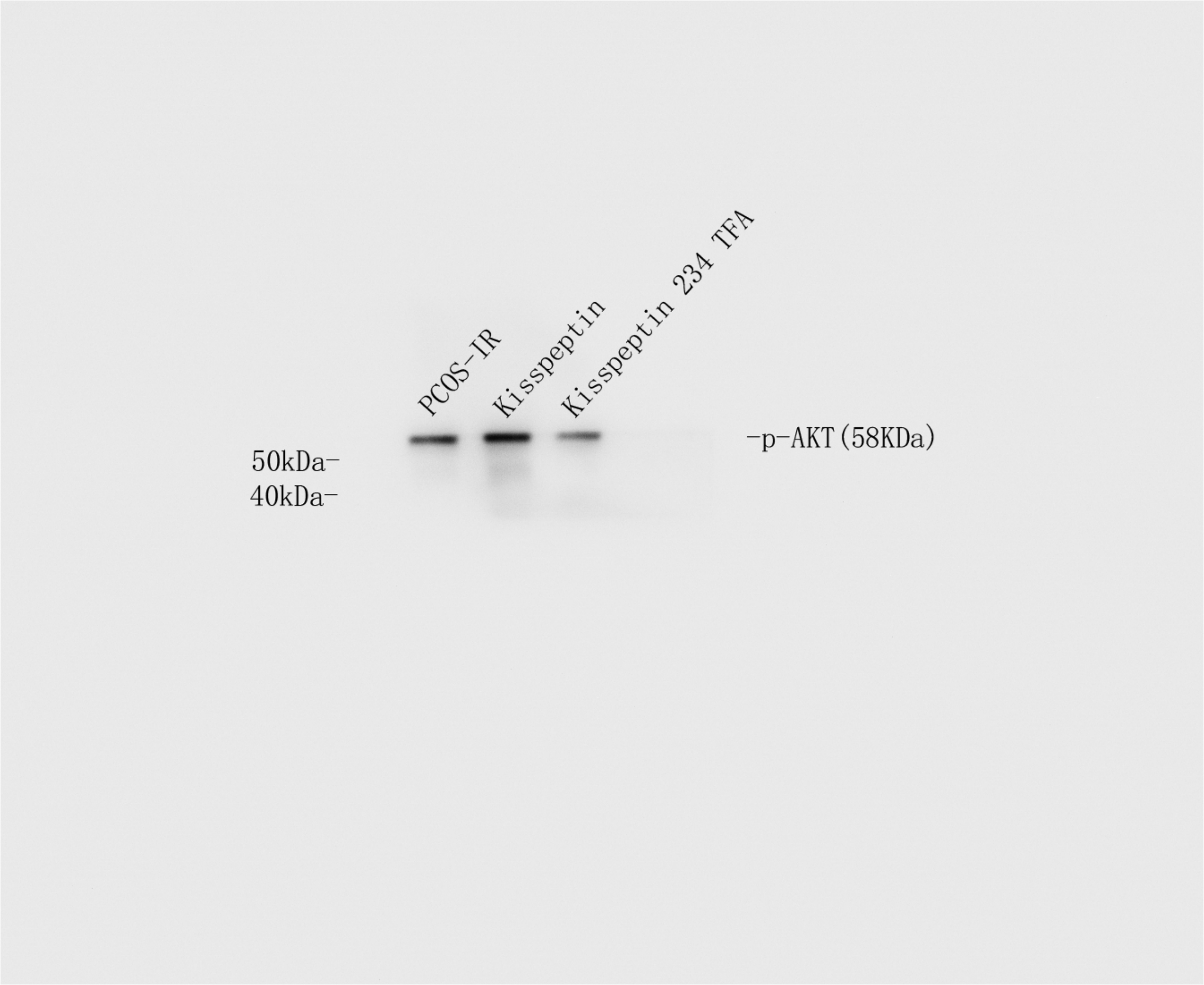

**AKT**

**Grouping from left to right:** PCOS-IR, Kisspeptin, Kisspeptin 234 TFA

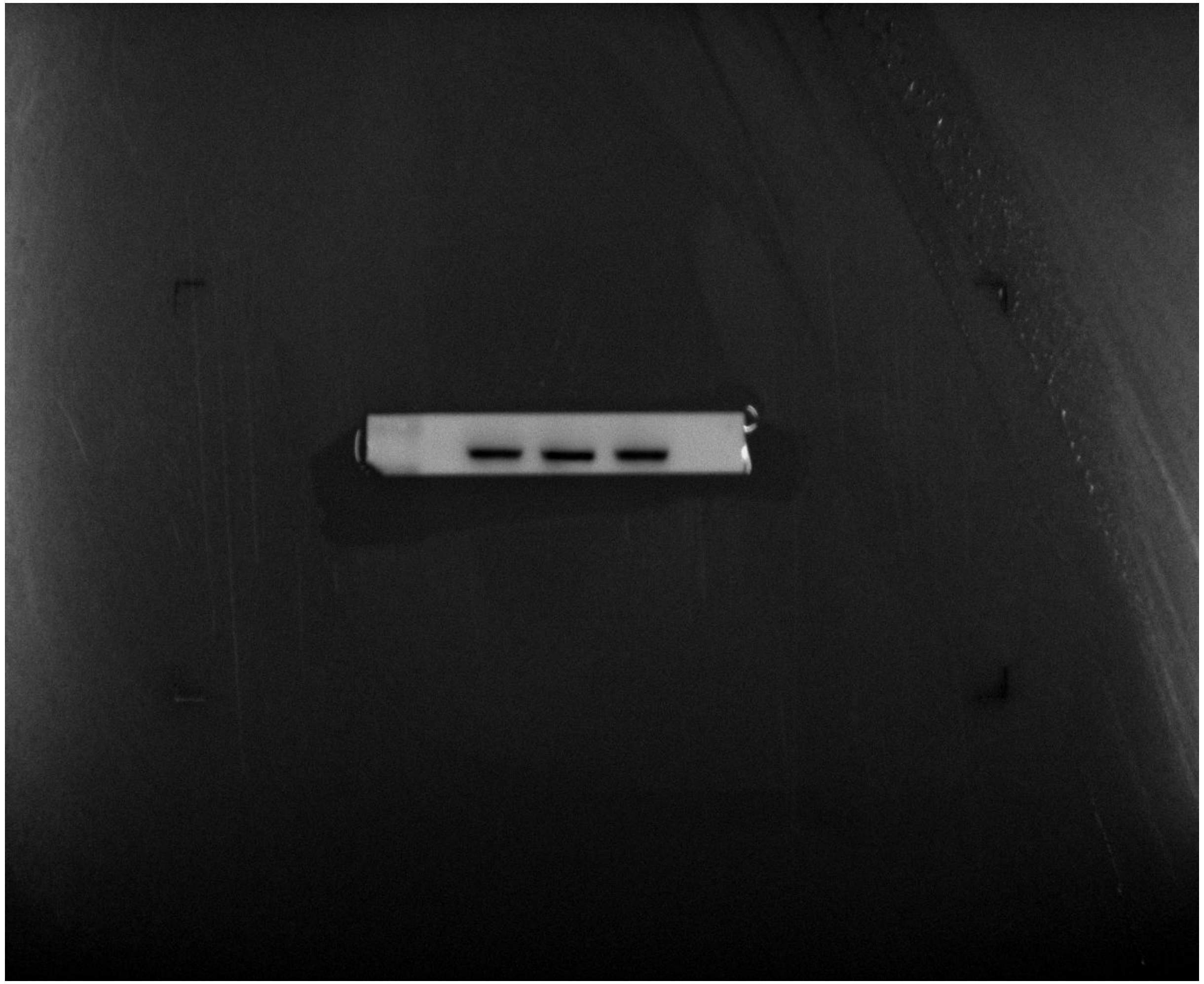

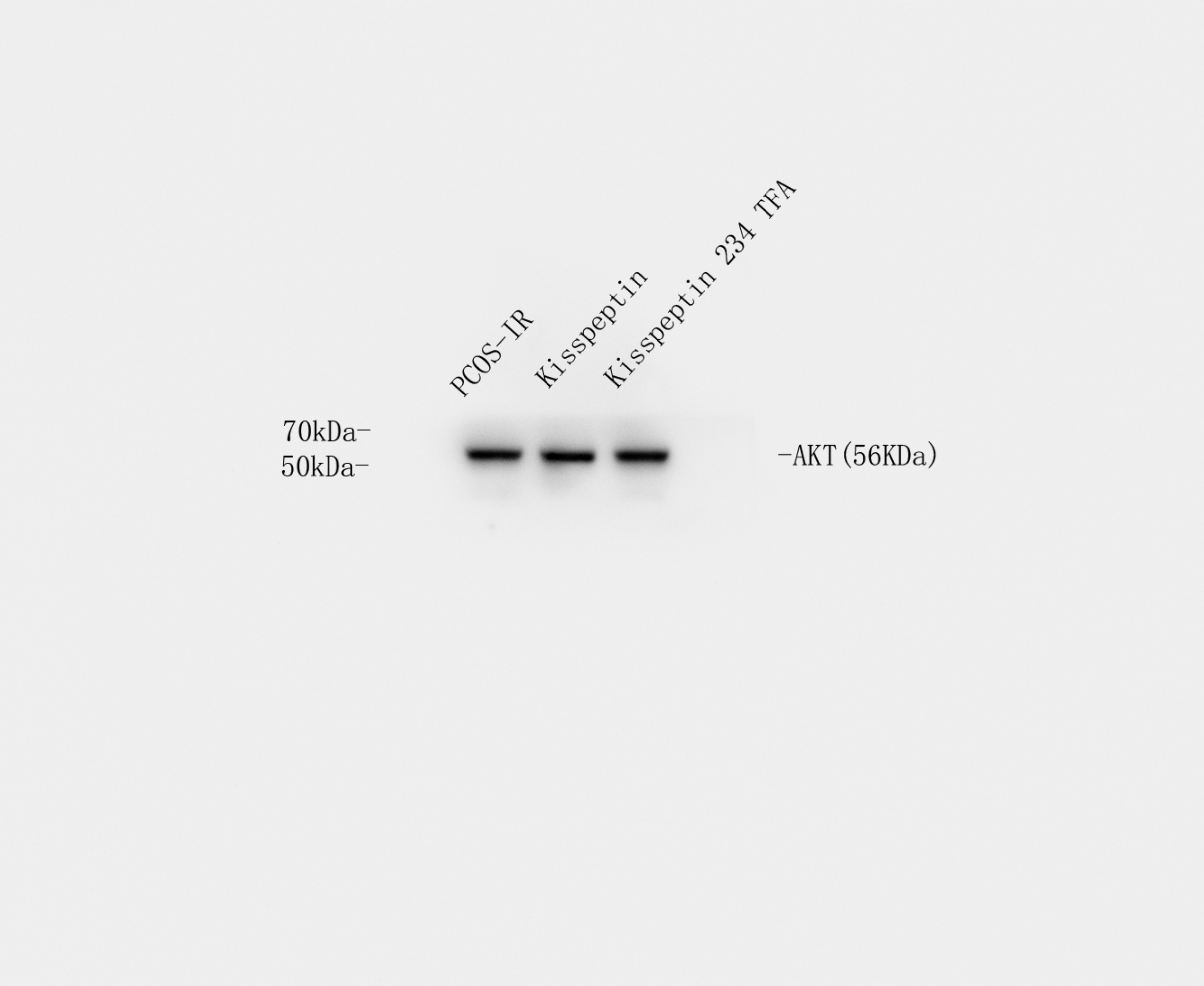

**GLUT4**
**Grouping from left to right:** PCOS-IR, Kisspeptin, Kisspeptin 234 TFA

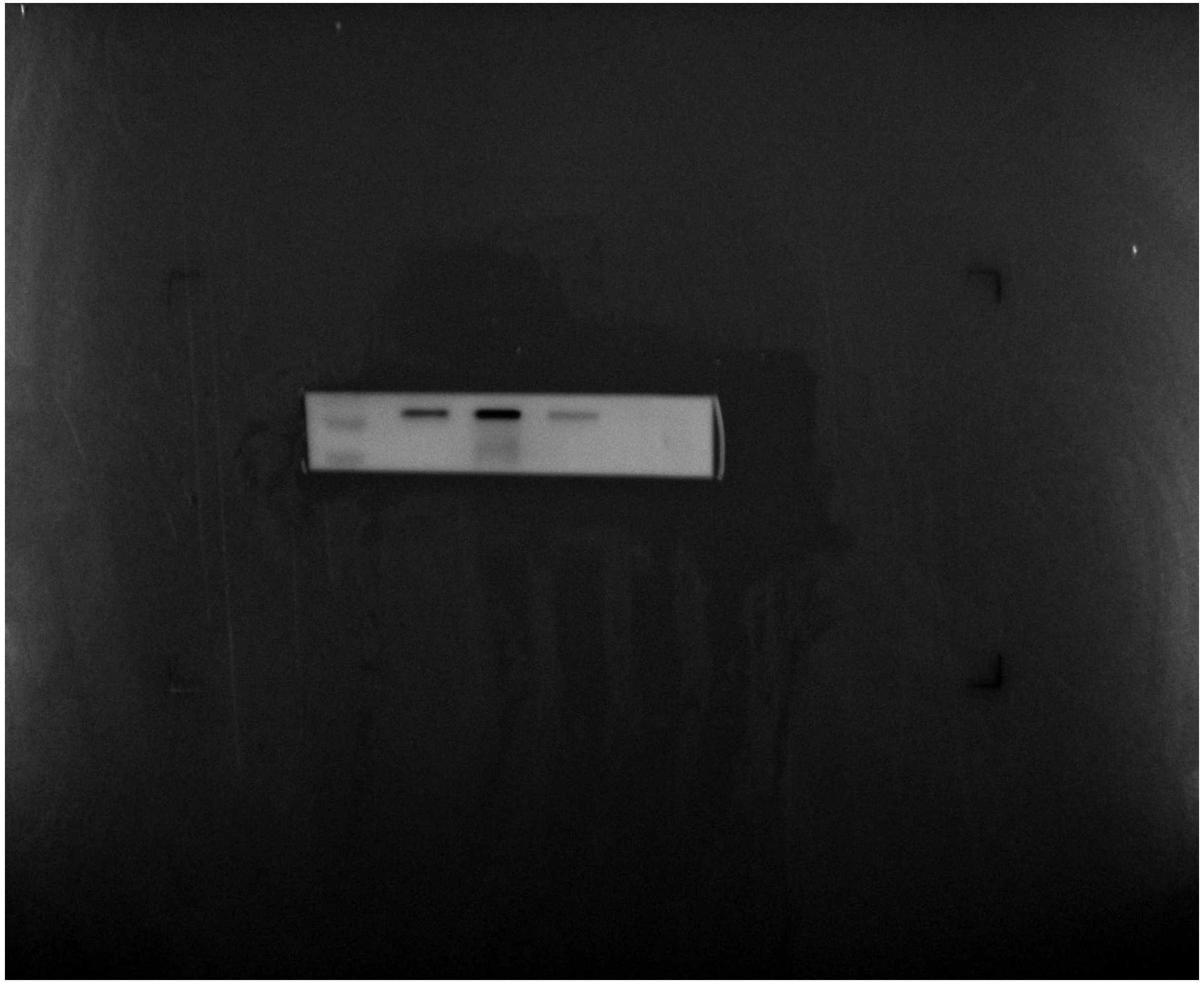

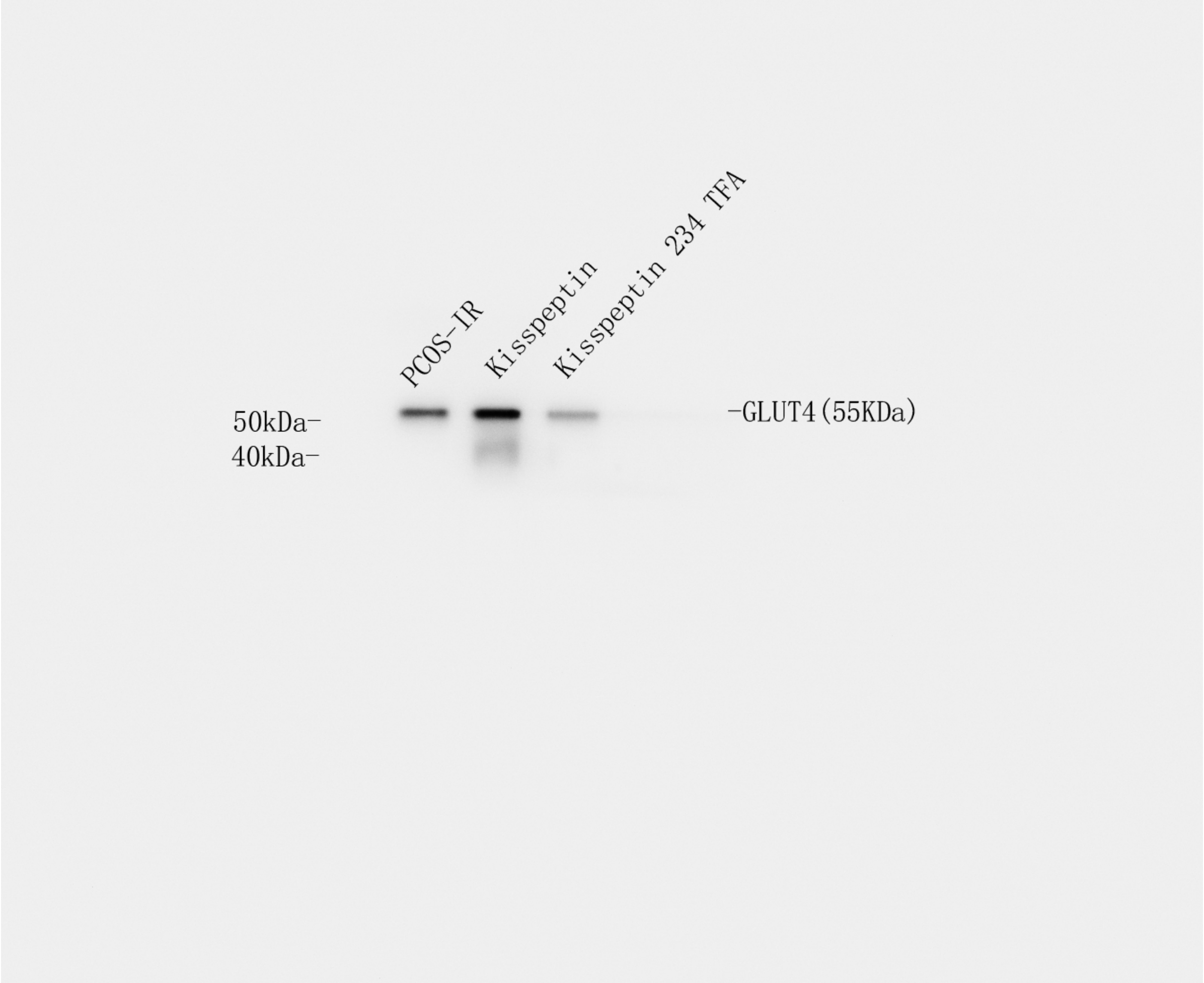

**p-PI3K**
**Grouping from left to right:** PCOS-IR, Kisspeptin, Kisspeptin 234 TFA

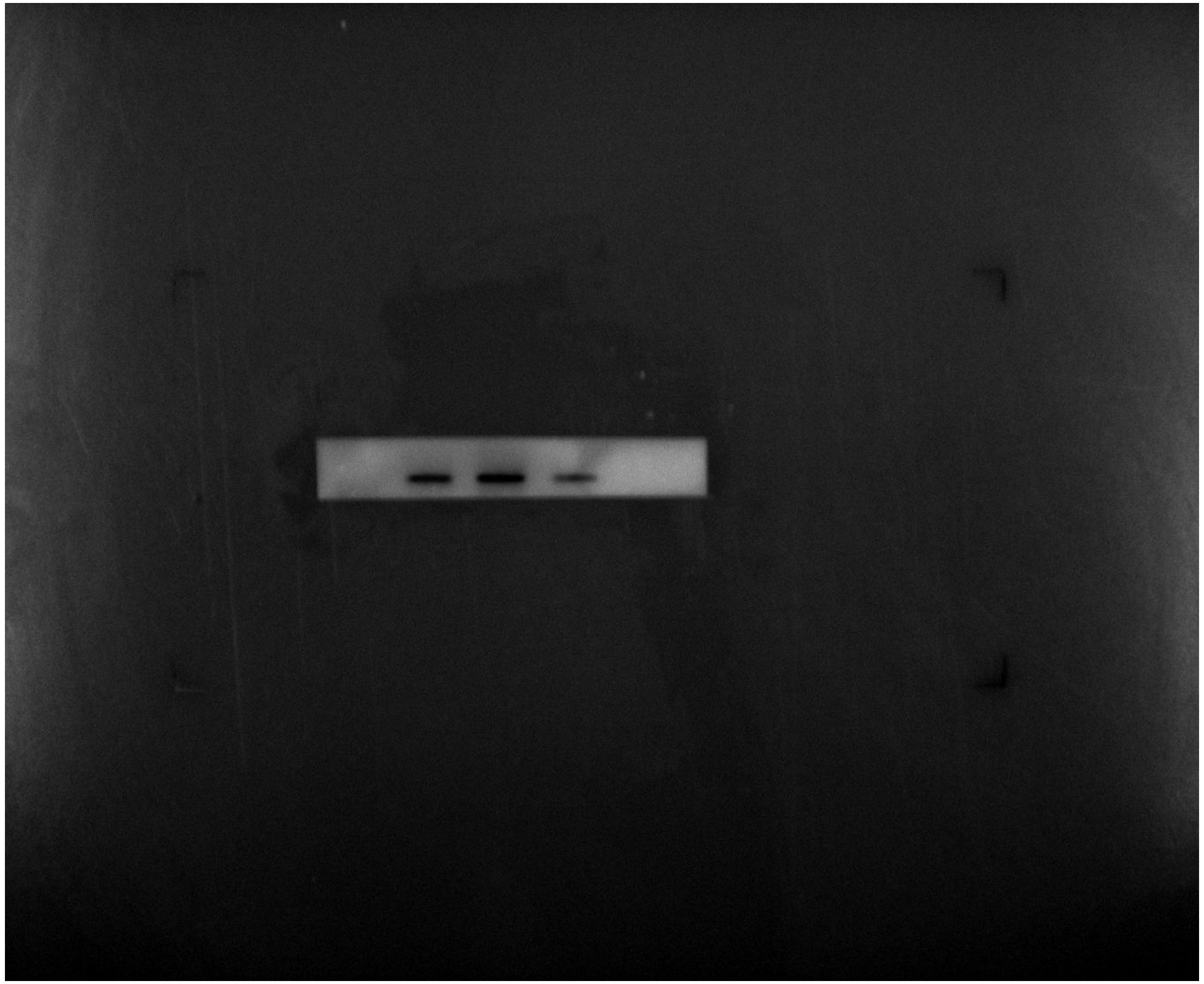

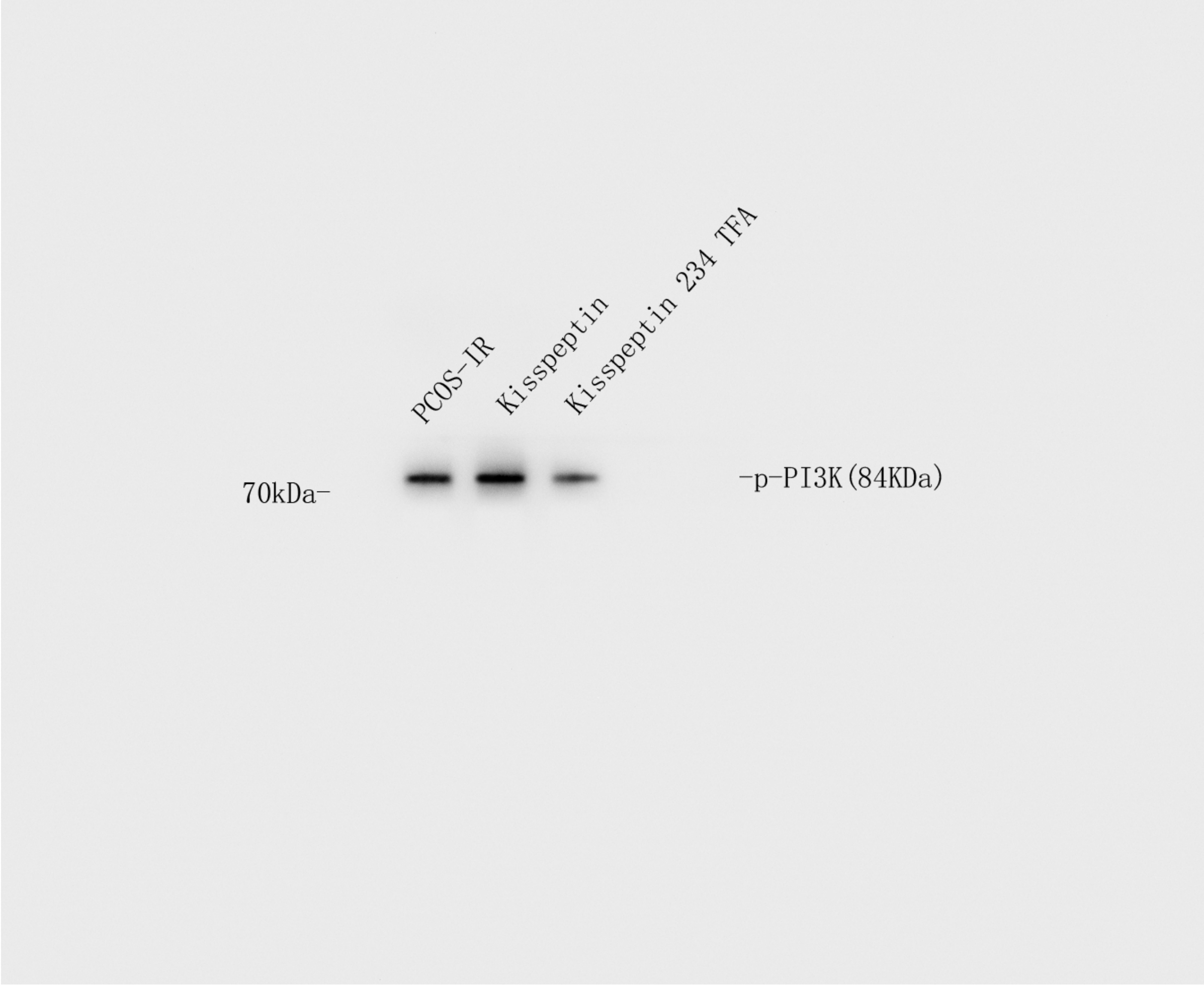

**PI3K**

**Grouping from left to right:** PCOS-IR, Kisspeptin, Kisspeptin 234 TFA

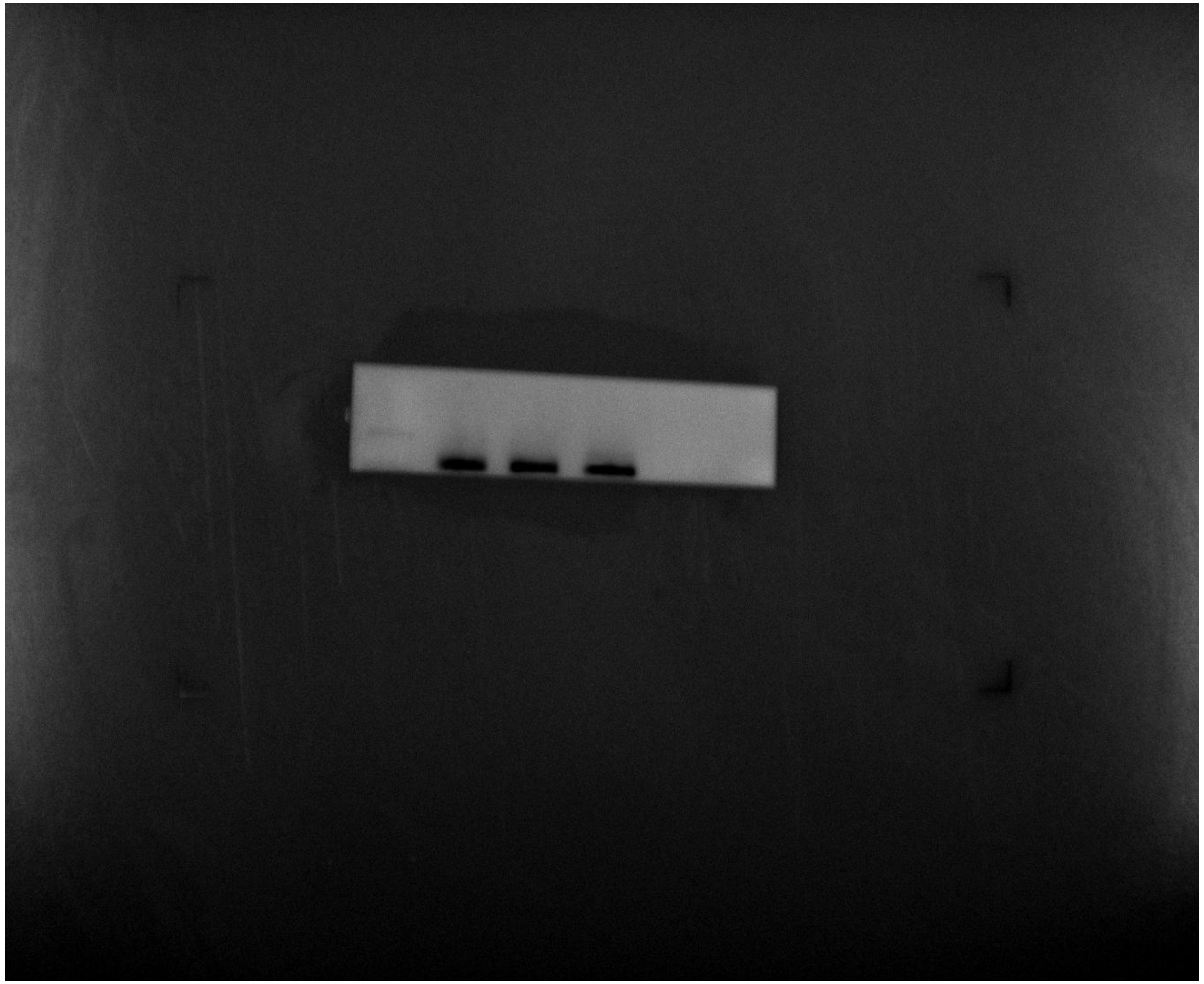

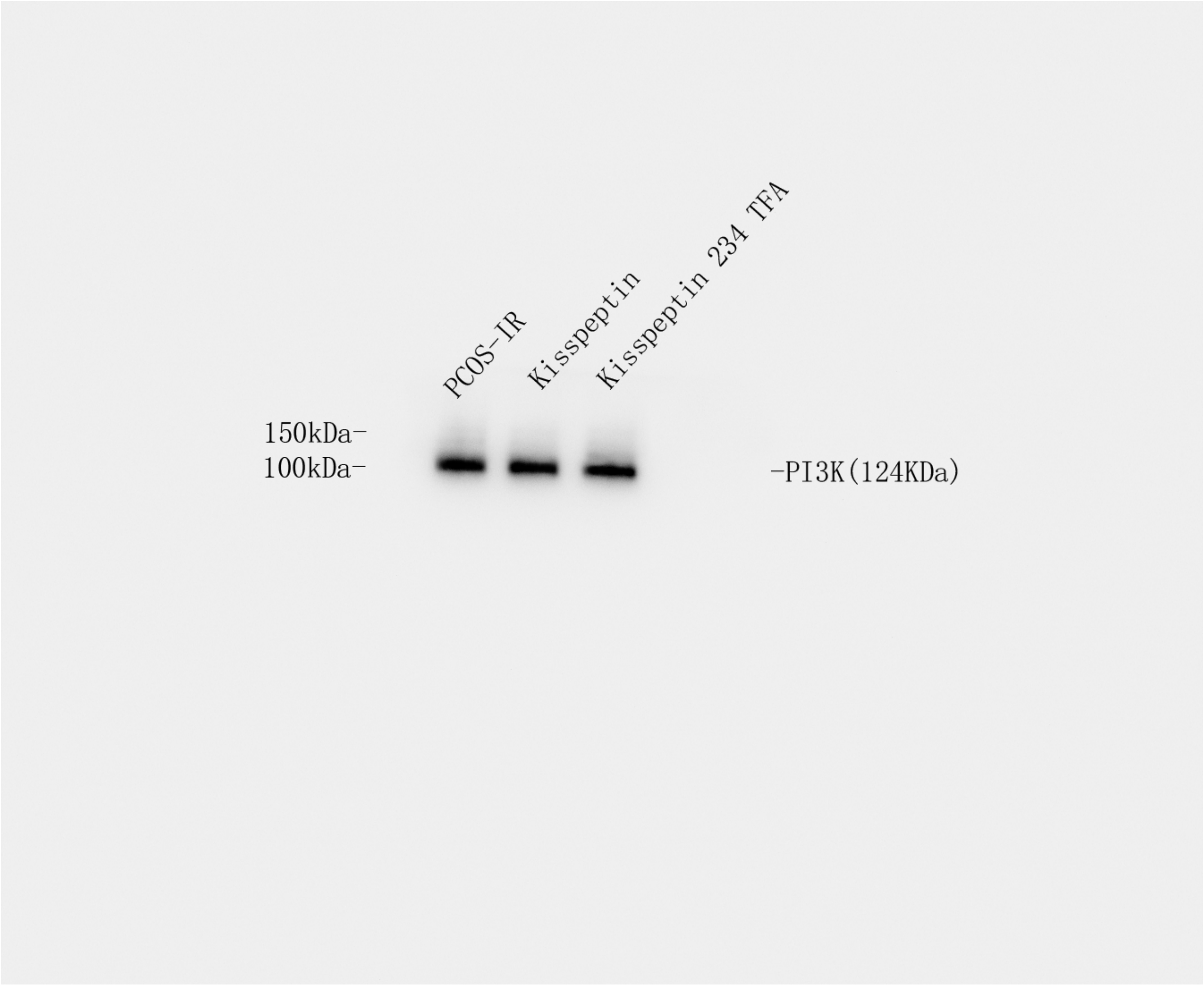

Supplement: S1 Data — (ZIP) [file pone.0342158.s001.zip › Supporting Information files/S1_raw_images.pdf]
