# Supplementary figures and images for: Kisspeptin improves local ovarian insulin resistance in PCOS by modulating the PI3K/AKT/GLUT4 signaling pathway
Source: PLoS One. 2026 Feb 2;21(2):e0342158. doi: 10.1371/journal.pone.0342158 (PMC12863573; doi:10.1371/journal.pone.0342158)

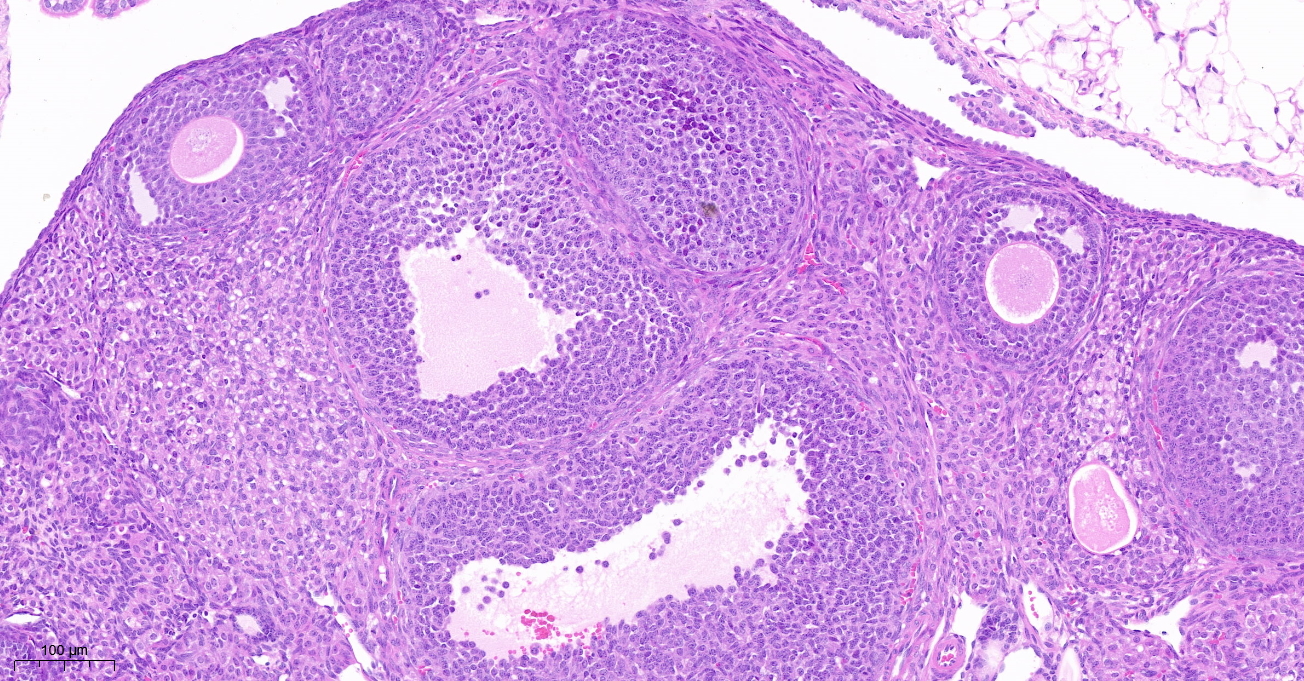

Supplement: S1 Data — (ZIP) [file pone.0342158.s001.zip › Supporting Information files/Raw data/FIG1/A-control.jpg]

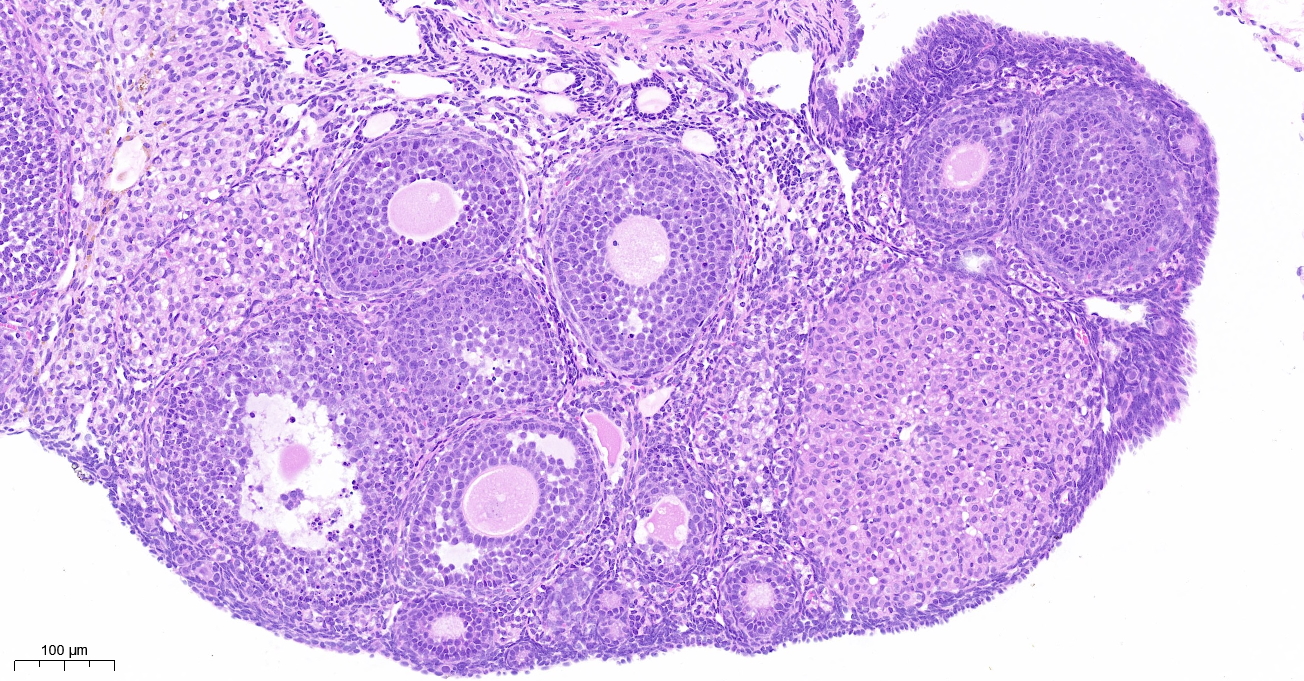

Supplement: S1 Data — (ZIP) [file pone.0342158.s001.zip › Supporting Information files/Raw data/FIG1/A-Model.jpg]

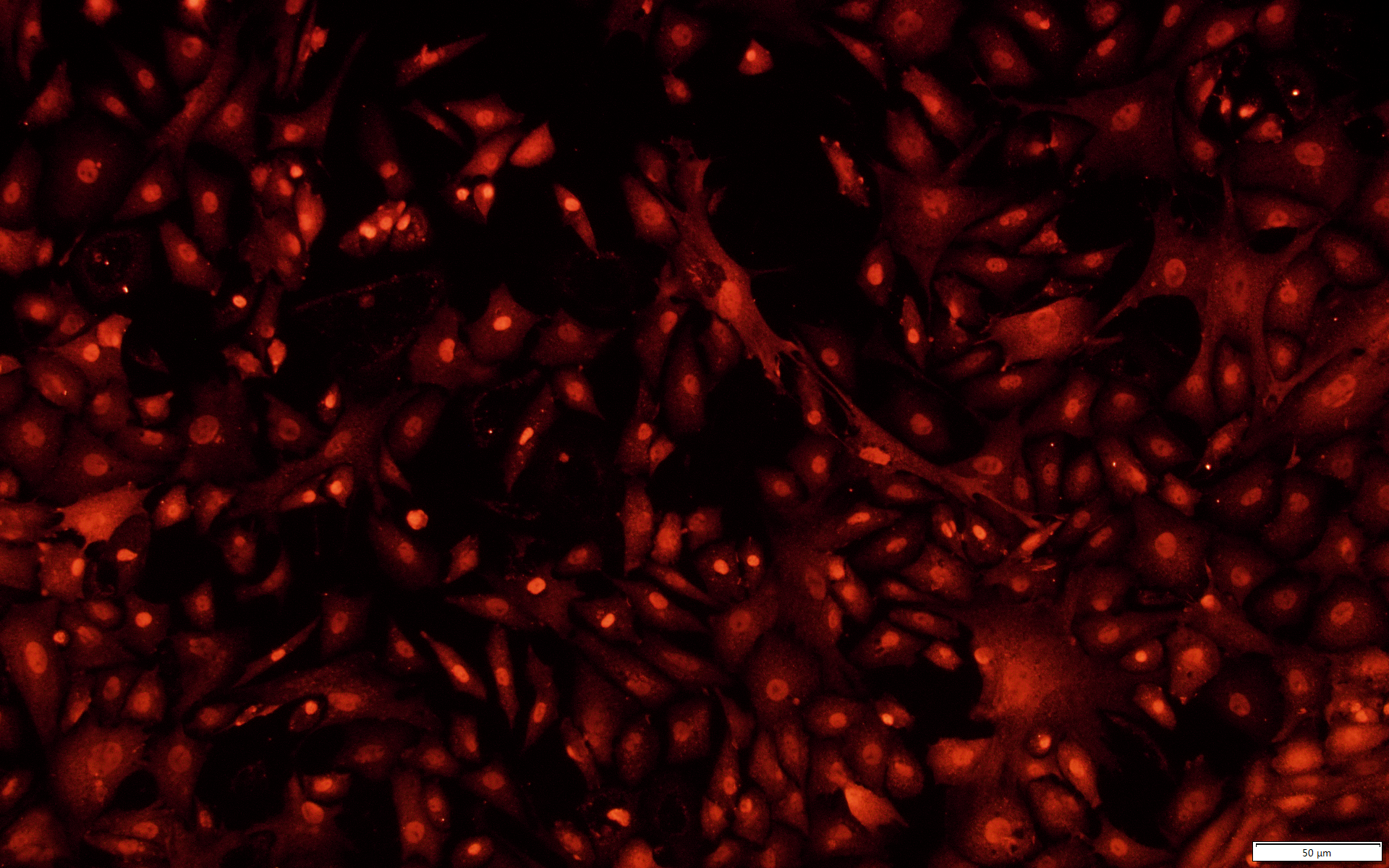

Supplement: S1 Data — (ZIP) [file pone.0342158.s001.zip › Supporting Information files/Raw data/FIG2/control 200x GLUT4.tif]

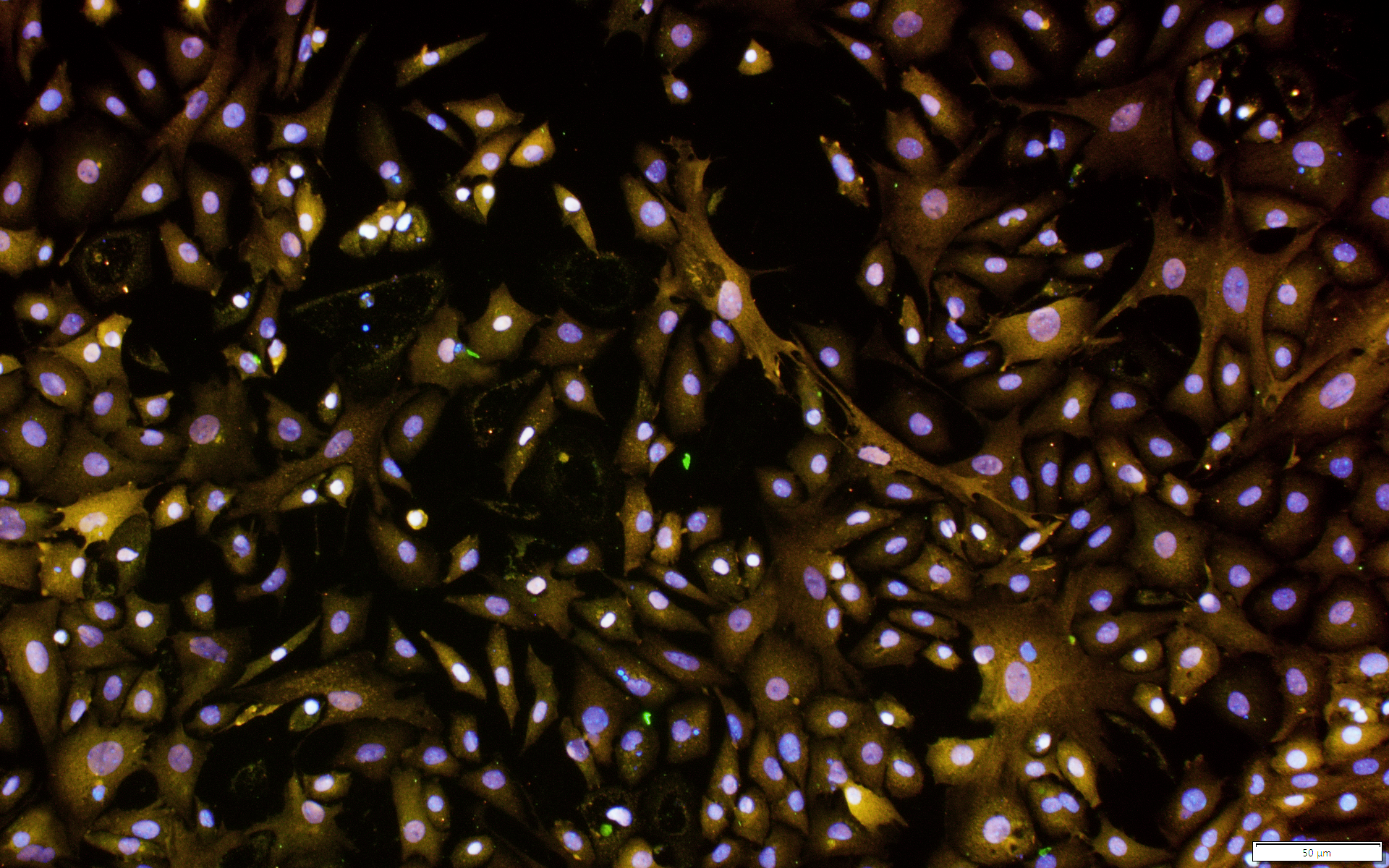

Supplement: S1 Data — (ZIP) [file pone.0342158.s001.zip › Supporting Information files/Raw data/FIG2/control 200x merge.tif]

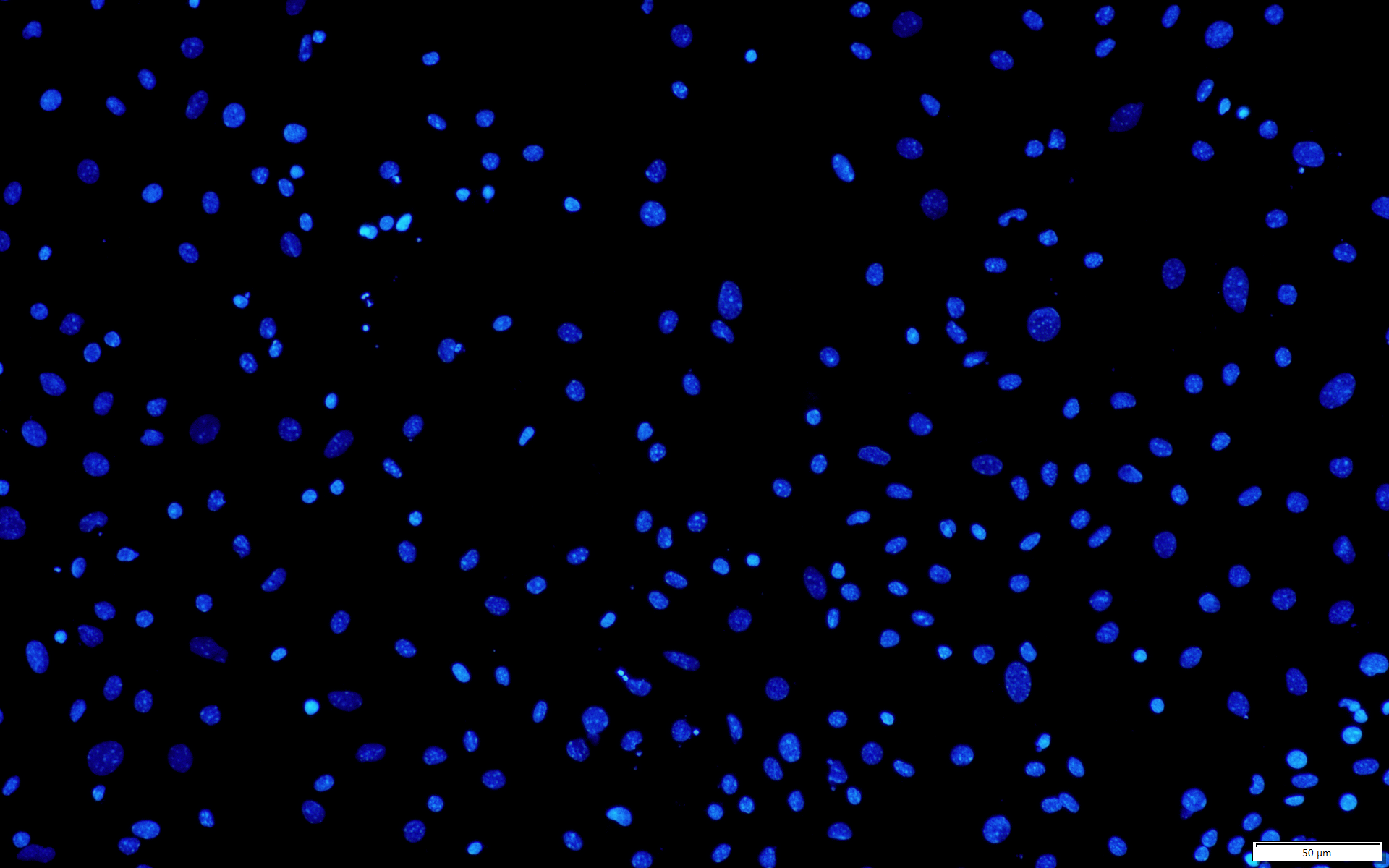

Supplement: S1 Data — (ZIP) [file pone.0342158.s001.zip › Supporting Information files/Raw data/FIG2/control 200x DAPI.tif]

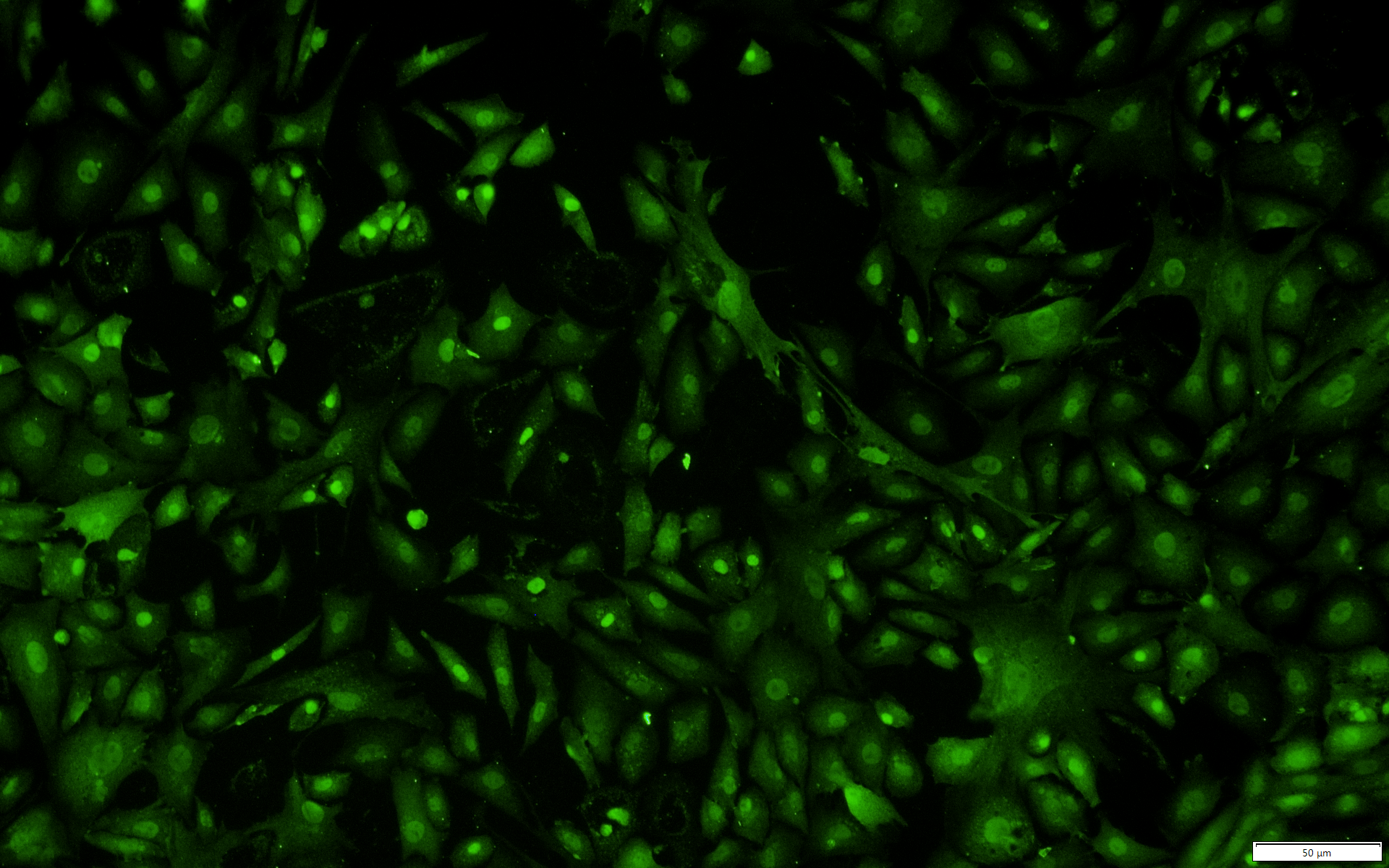

Supplement: S1 Data — (ZIP) [file pone.0342158.s001.zip › Supporting Information files/Raw data/FIG2/control 200x Kisspeptin.tif]

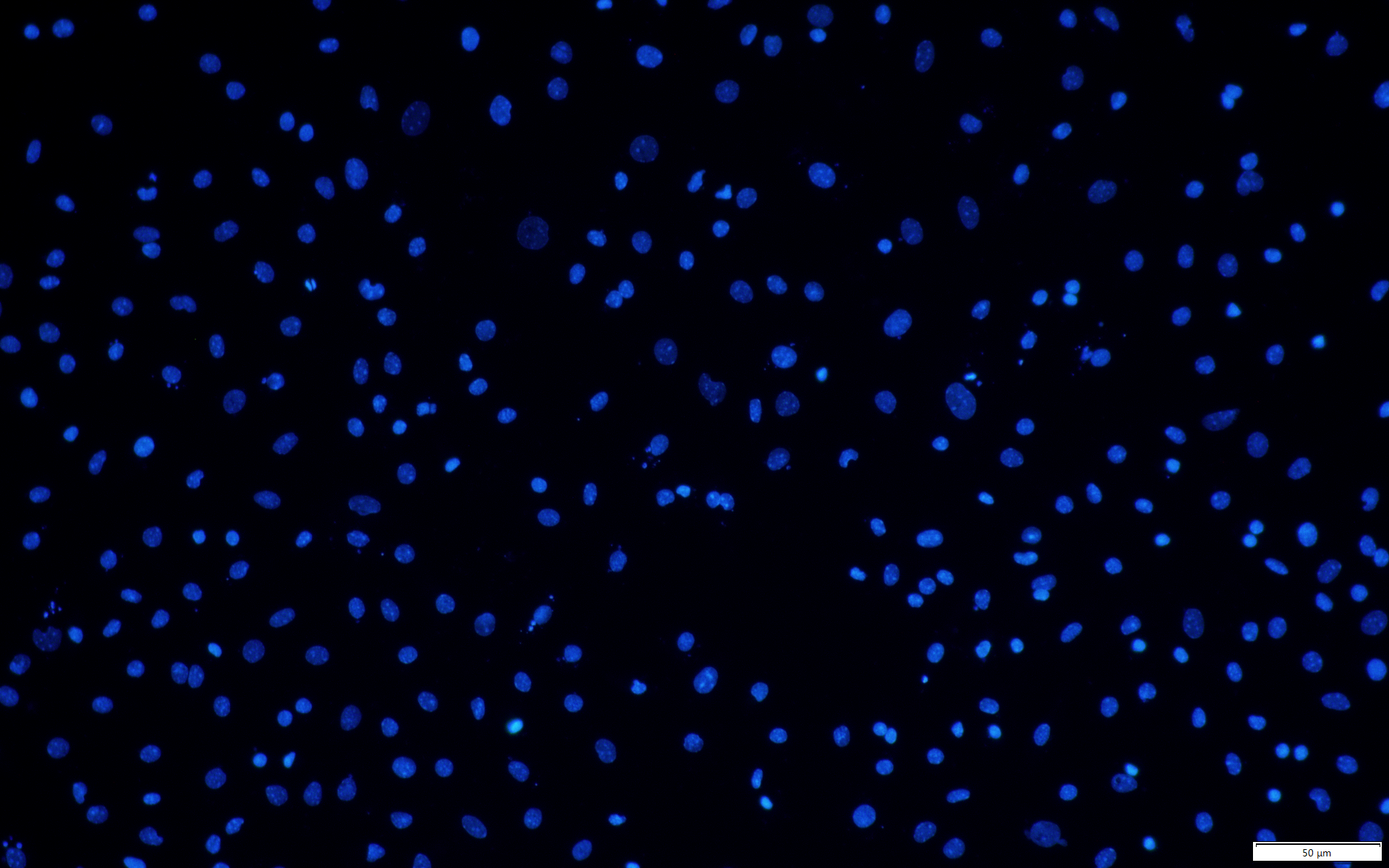

Supplement: S1 Data — (ZIP) [file pone.0342158.s001.zip › Supporting Information files/Raw data/FIG2/PCOS-IR 200x DAPI.tif]

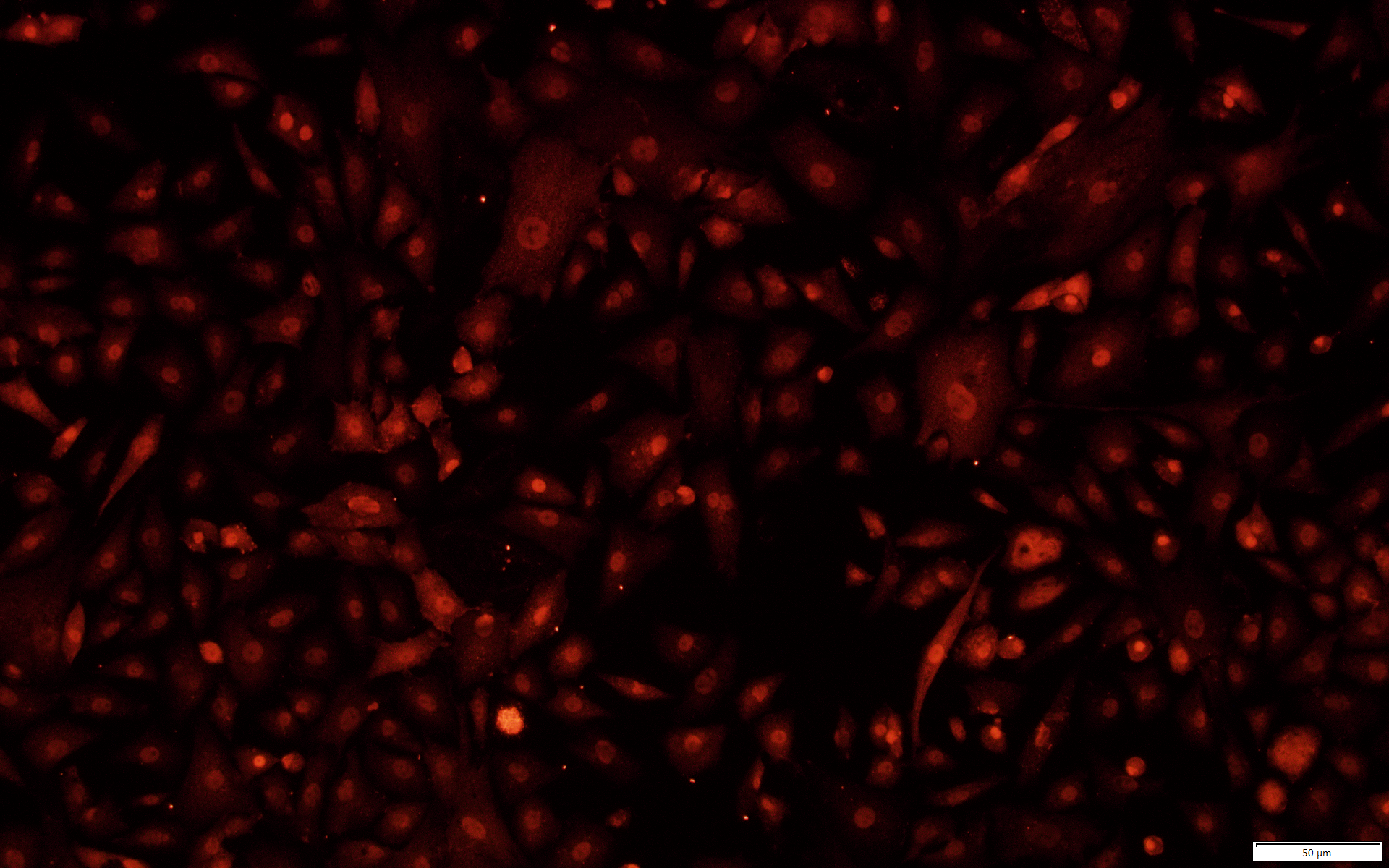

Supplement: S1 Data — (ZIP) [file pone.0342158.s001.zip › Supporting Information files/Raw data/FIG2/PCOS-IR 200x GLUT4.tif]

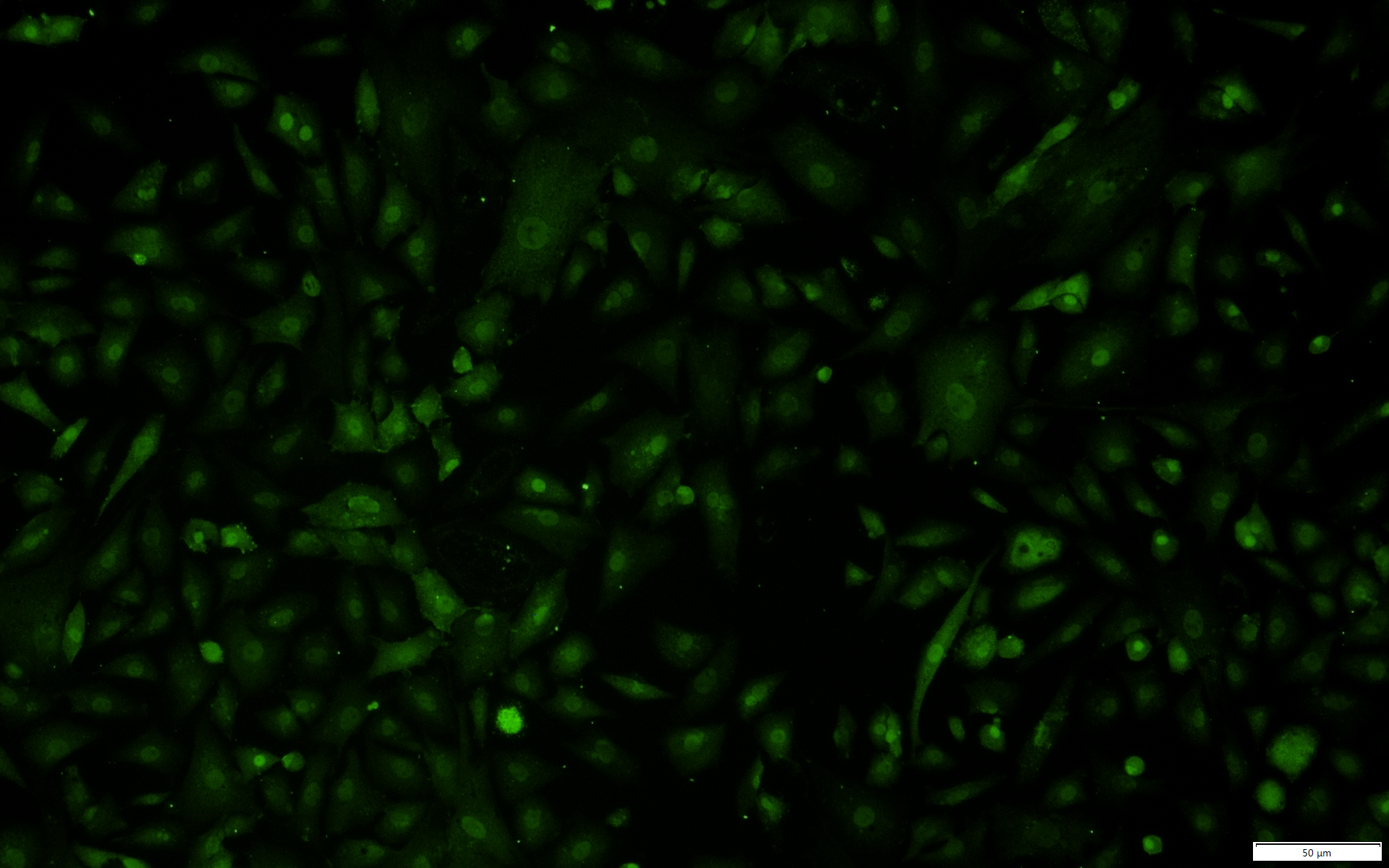

Supplement: S1 Data — (ZIP) [file pone.0342158.s001.zip › Supporting Information files/Raw data/FIG2/PCOS-IR 200x Kisspeptin.tif]

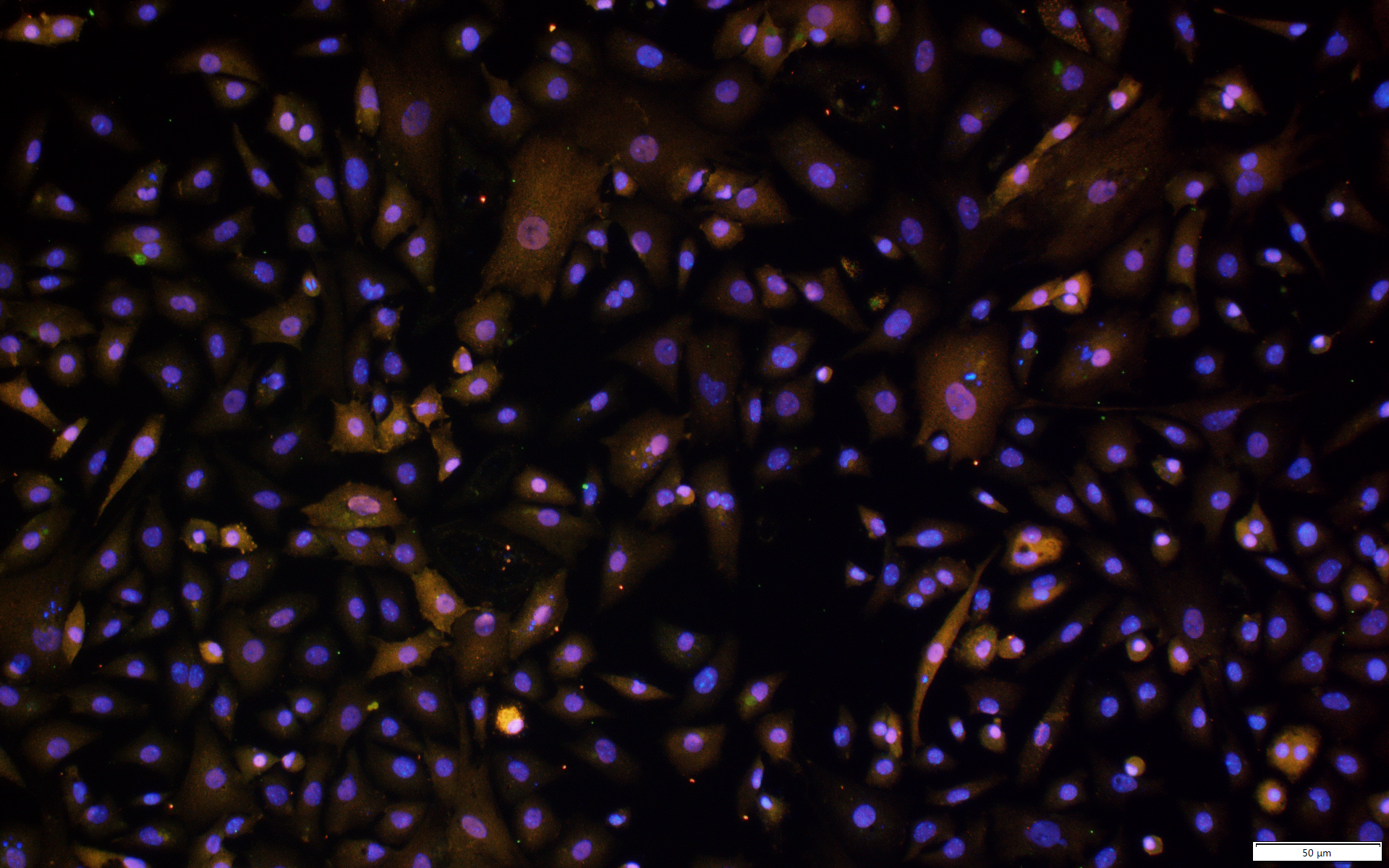

Supplement: S1 Data — (ZIP) [file pone.0342158.s001.zip › Supporting Information files/Raw data/FIG2/PCOS-IR 200x merge.tif]

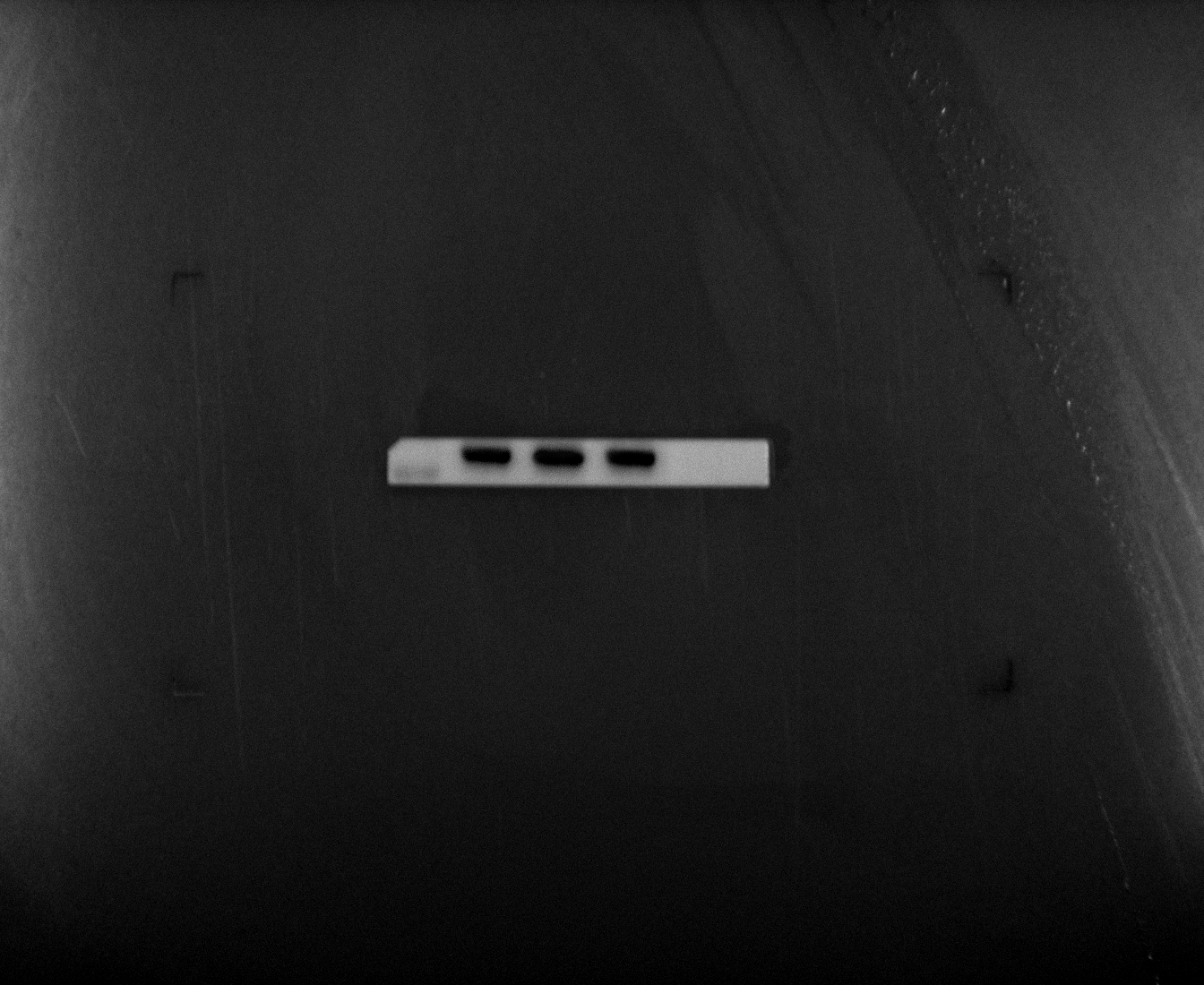

Supplement: S1 Data — (ZIP) [file pone.0342158.s001.zip › Supporting Information files/Raw data/FIG3/actin merge.tif]

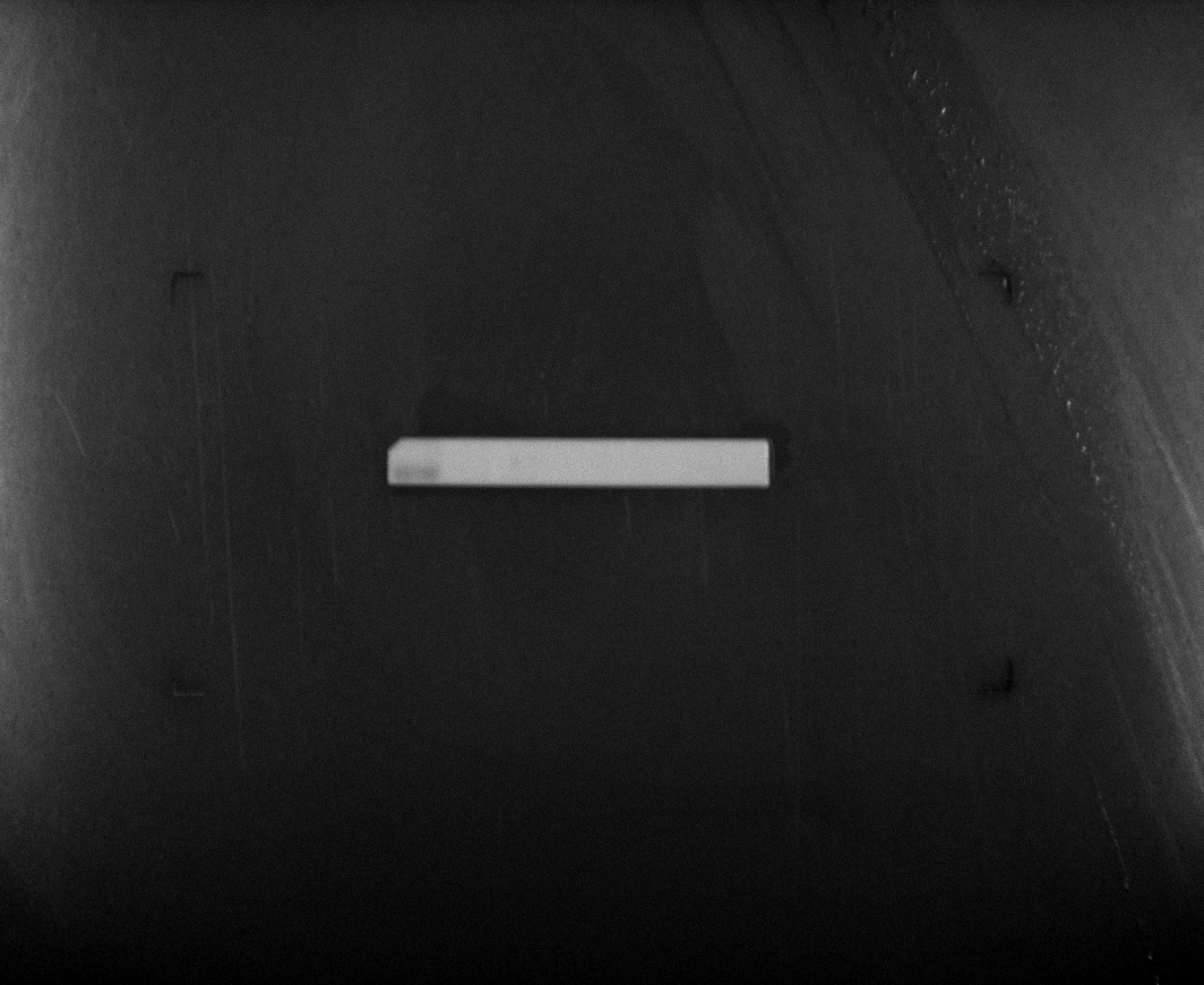

Supplement: S1 Data — (ZIP) [file pone.0342158.s001.zip › Supporting Information files/Raw data/FIG3/actin white light.Tif]

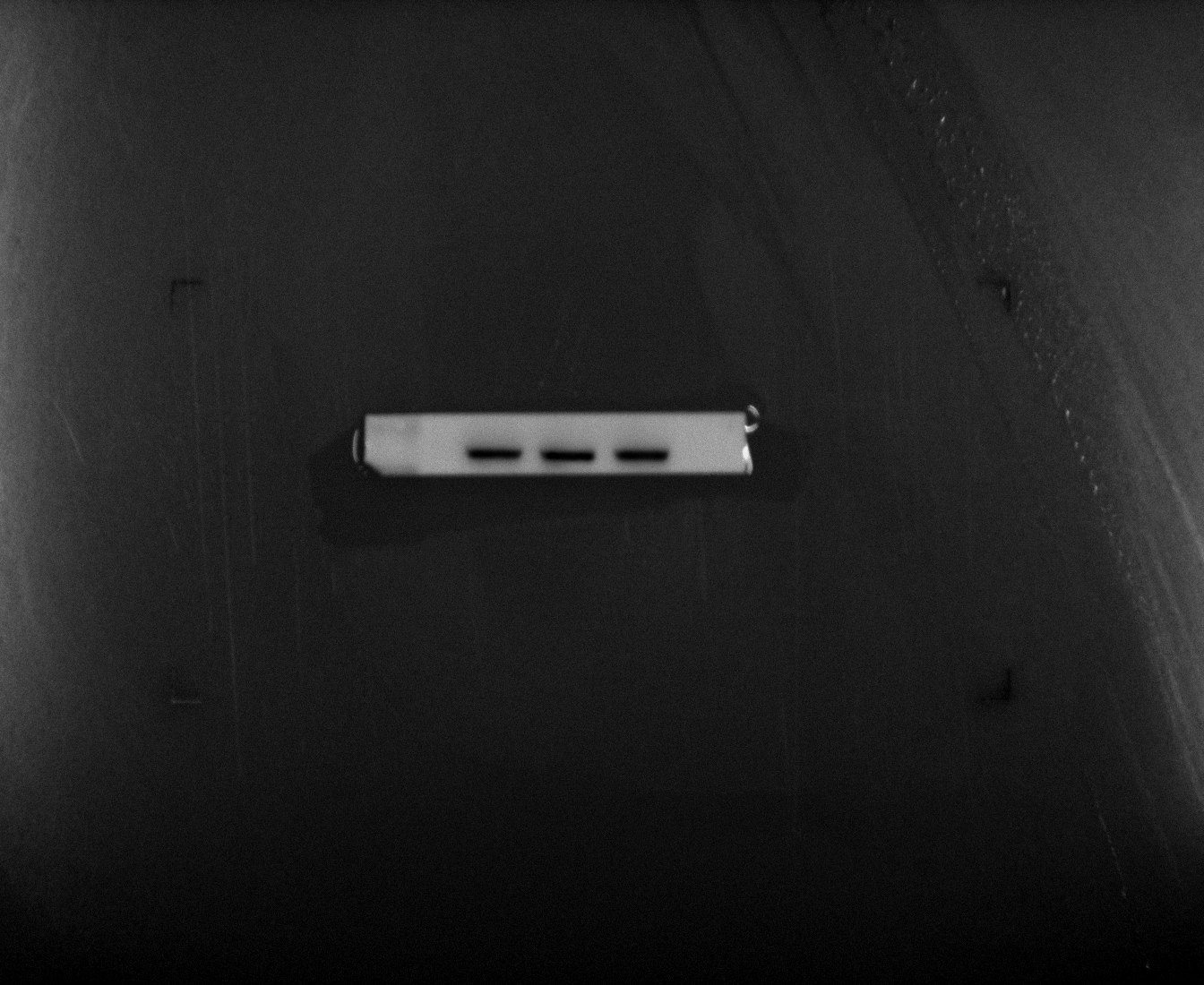

Supplement: S1 Data — (ZIP) [file pone.0342158.s001.zip › Supporting Information files/Raw data/FIG3/AKT merge.tif]

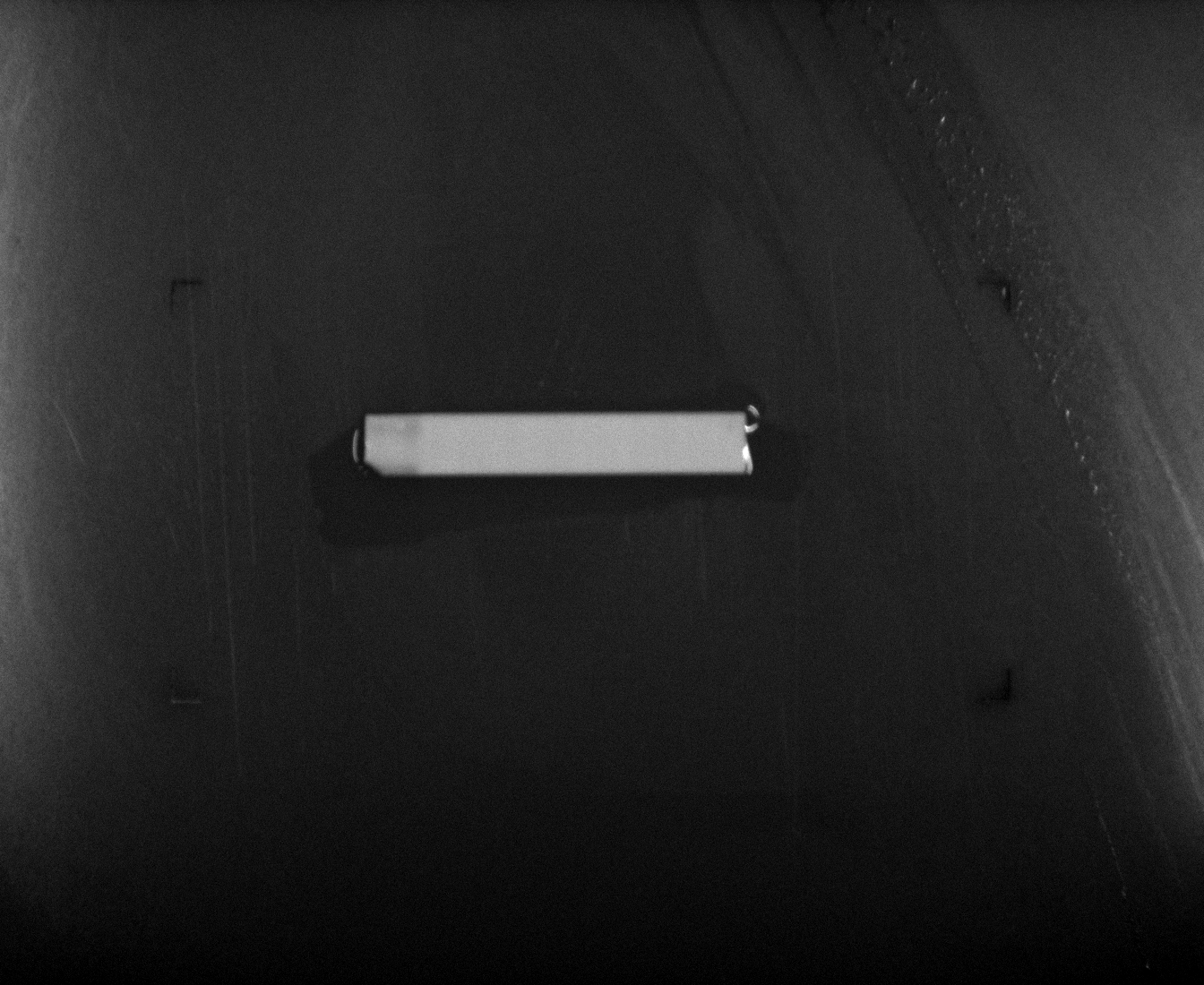

Supplement: S1 Data — (ZIP) [file pone.0342158.s001.zip › Supporting Information files/Raw data/FIG3/AKT white light.Tif]

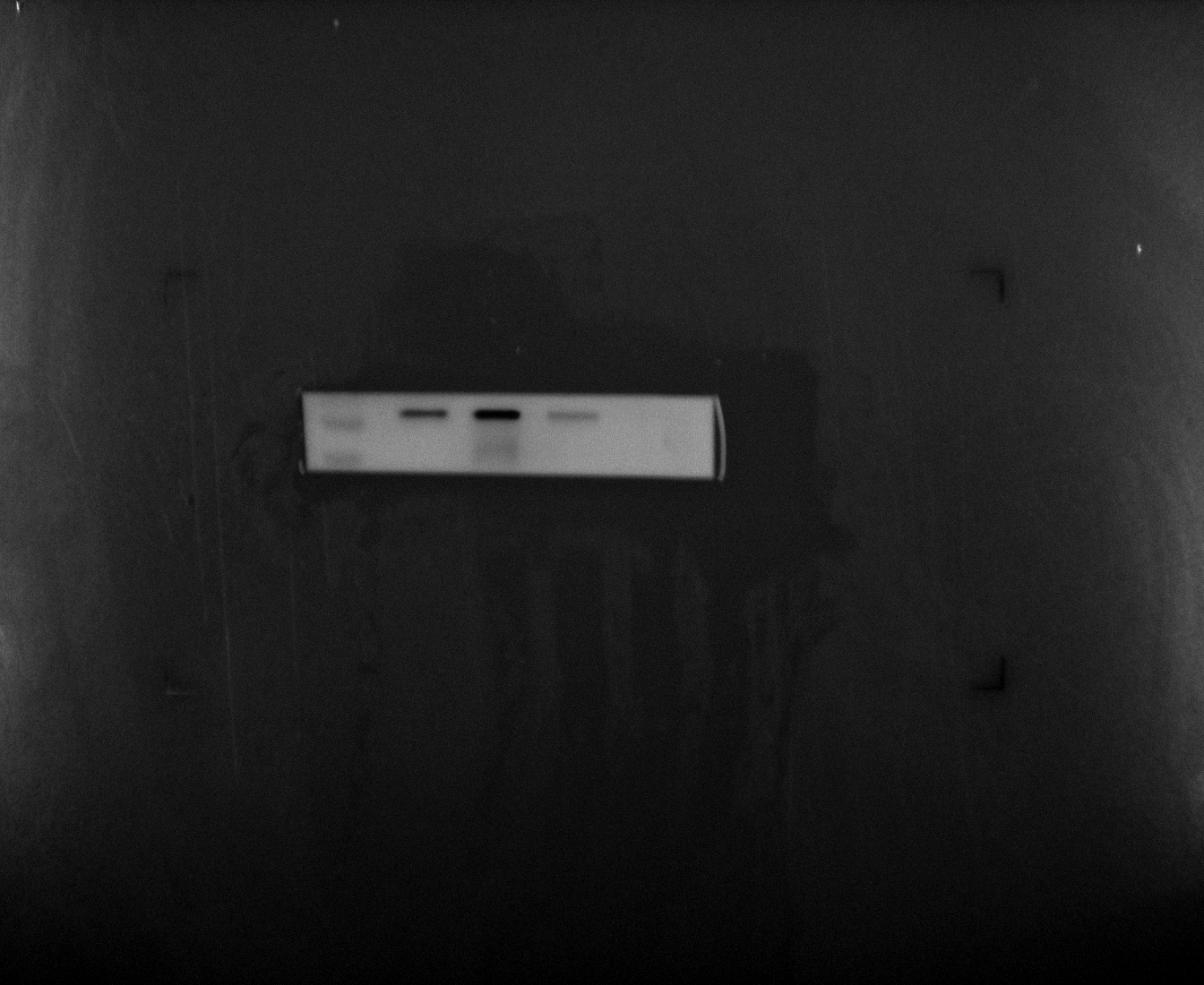

Supplement: S1 Data — (ZIP) [file pone.0342158.s001.zip › Supporting Information files/Raw data/FIG3/GLUT4 merge.tif]

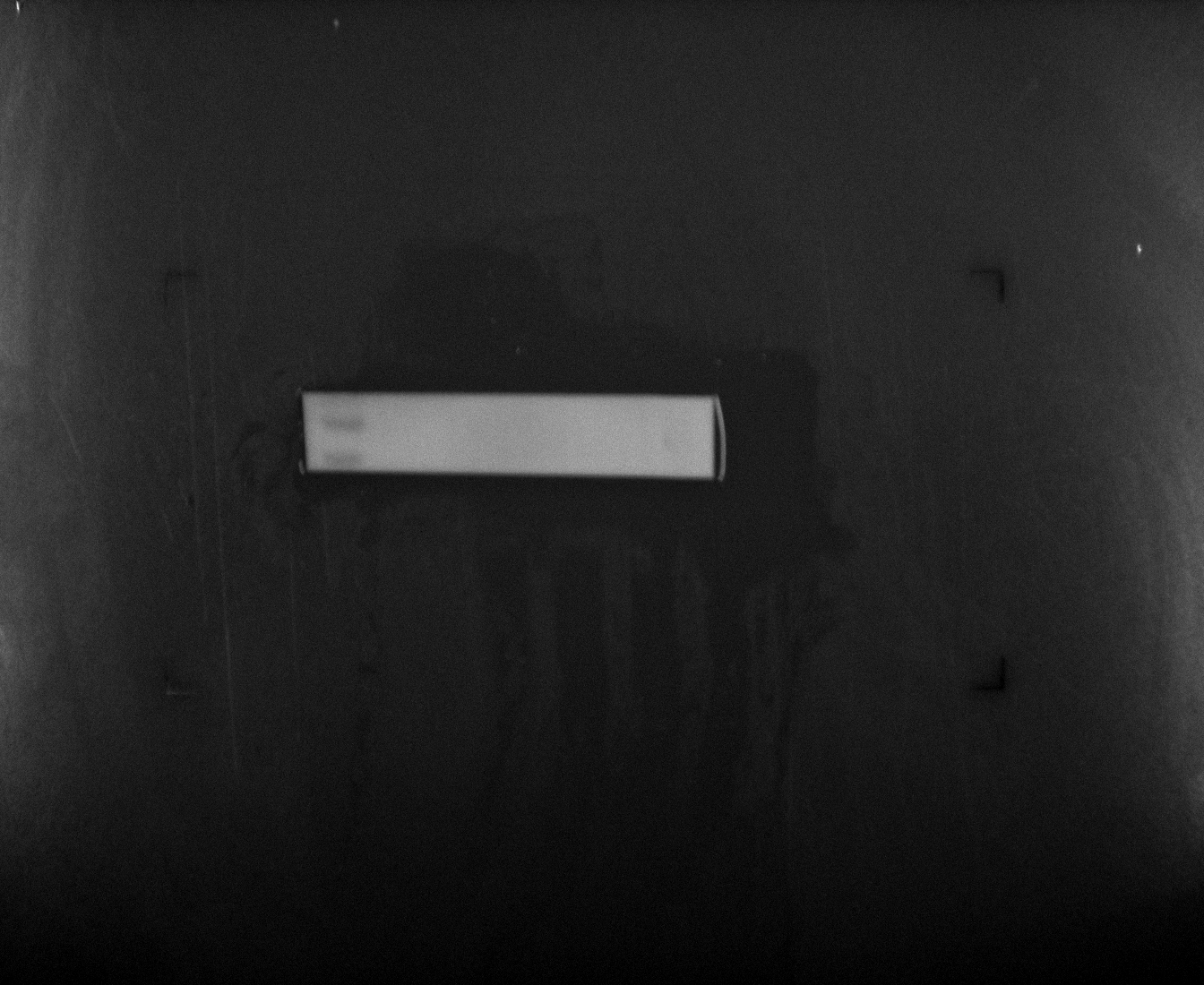

Supplement: S1 Data — (ZIP) [file pone.0342158.s001.zip › Supporting Information files/Raw data/FIG3/GLUT4 white light.Tif]

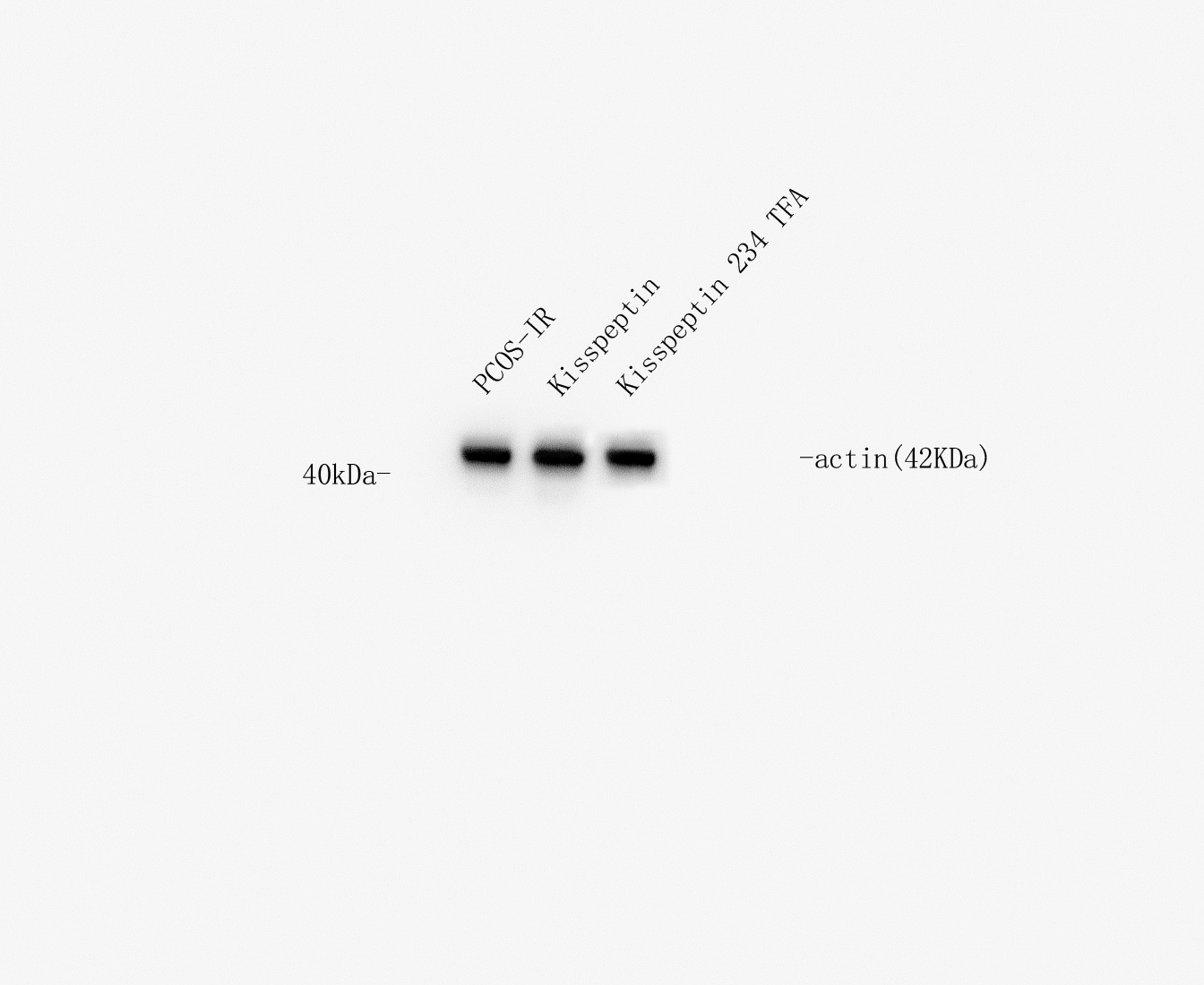

Supplement: S1 Data — (ZIP) [file pone.0342158.s001.zip › Supporting Information files/Raw data/FIG3/labeled blot image-actin(1).tif]

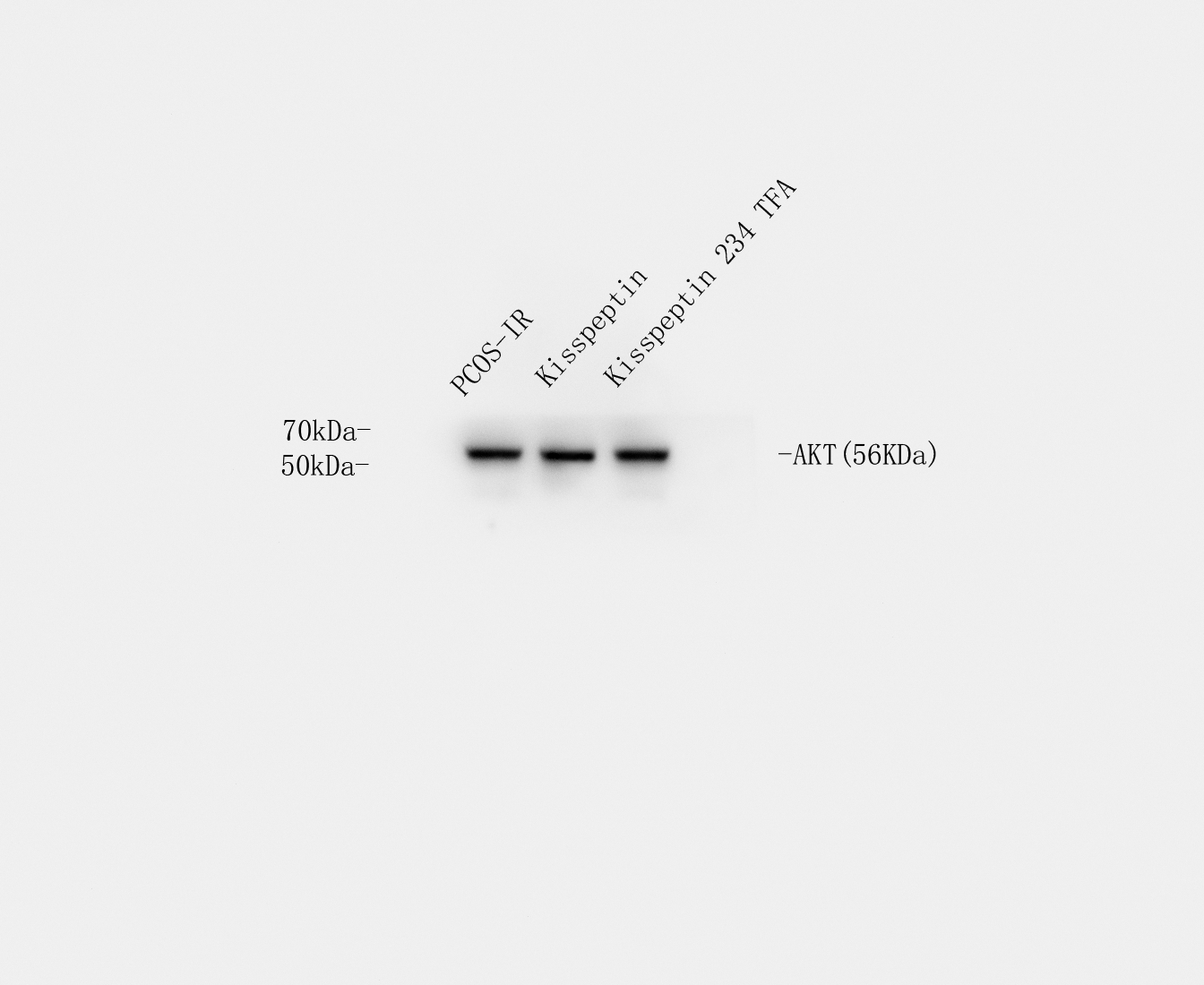

Supplement: S1 Data — (ZIP) [file pone.0342158.s001.zip › Supporting Information files/Raw data/FIG3/labeled blot image-AKT(1).tif]

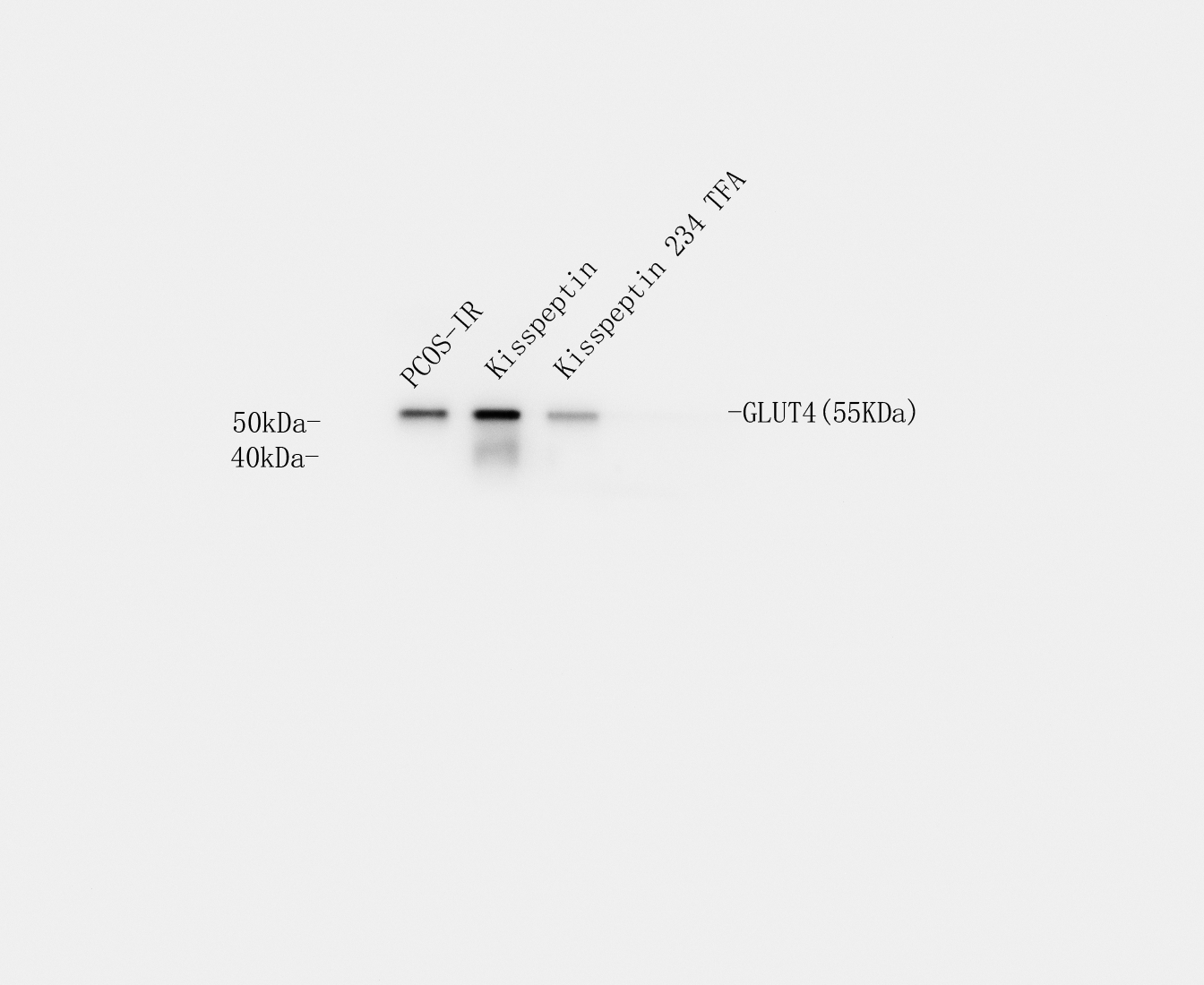

Supplement: S1 Data — (ZIP) [file pone.0342158.s001.zip › Supporting Information files/Raw data/FIG3/labeled blot image-GLUT4(1).tif]

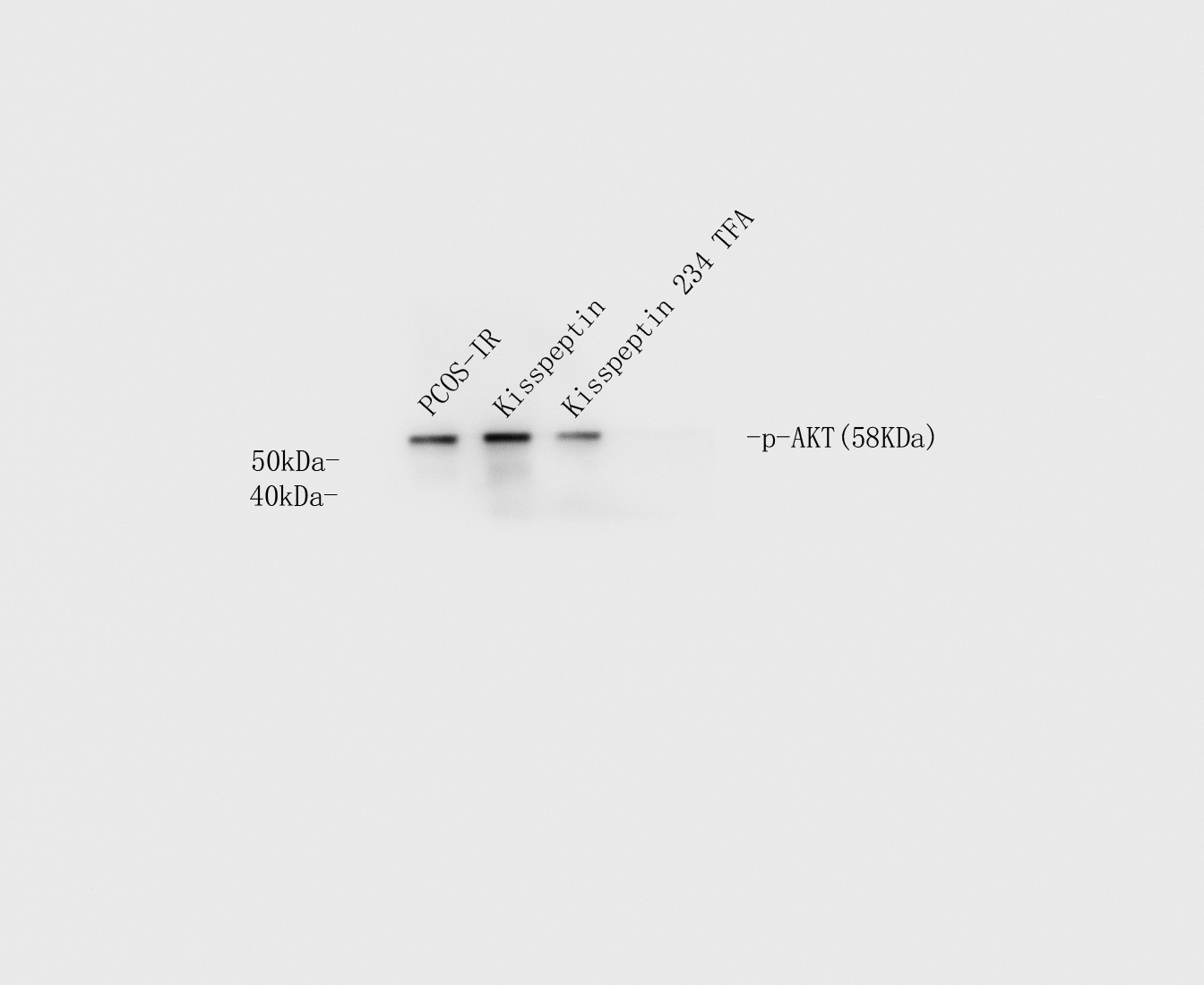

Supplement: S1 Data — (ZIP) [file pone.0342158.s001.zip › Supporting Information files/Raw data/FIG3/labeled blot image-p-AKT(1).tif]

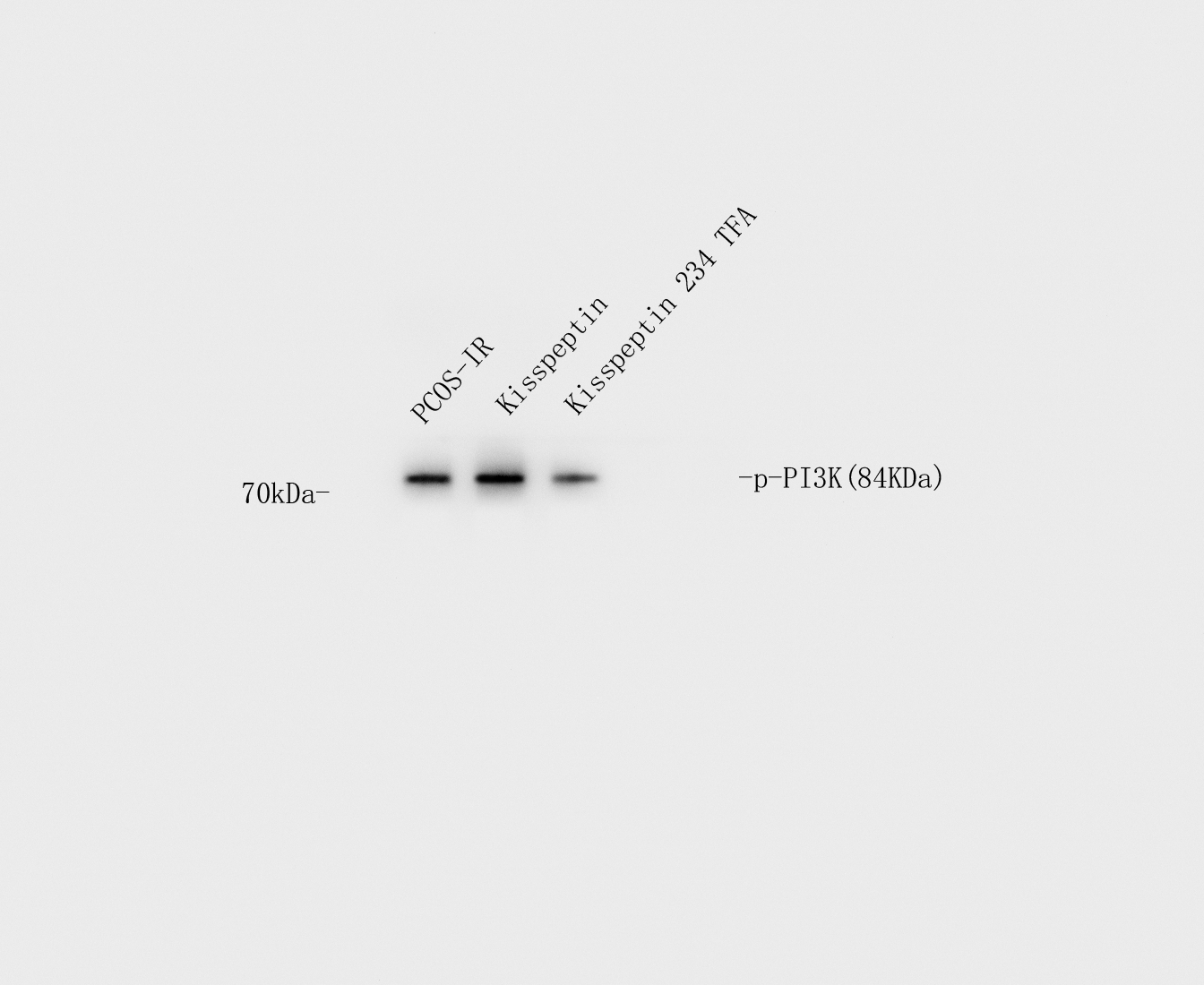

Supplement: S1 Data — (ZIP) [file pone.0342158.s001.zip › Supporting Information files/Raw data/FIG3/labeled blot image-P-PI3K(1).tif]

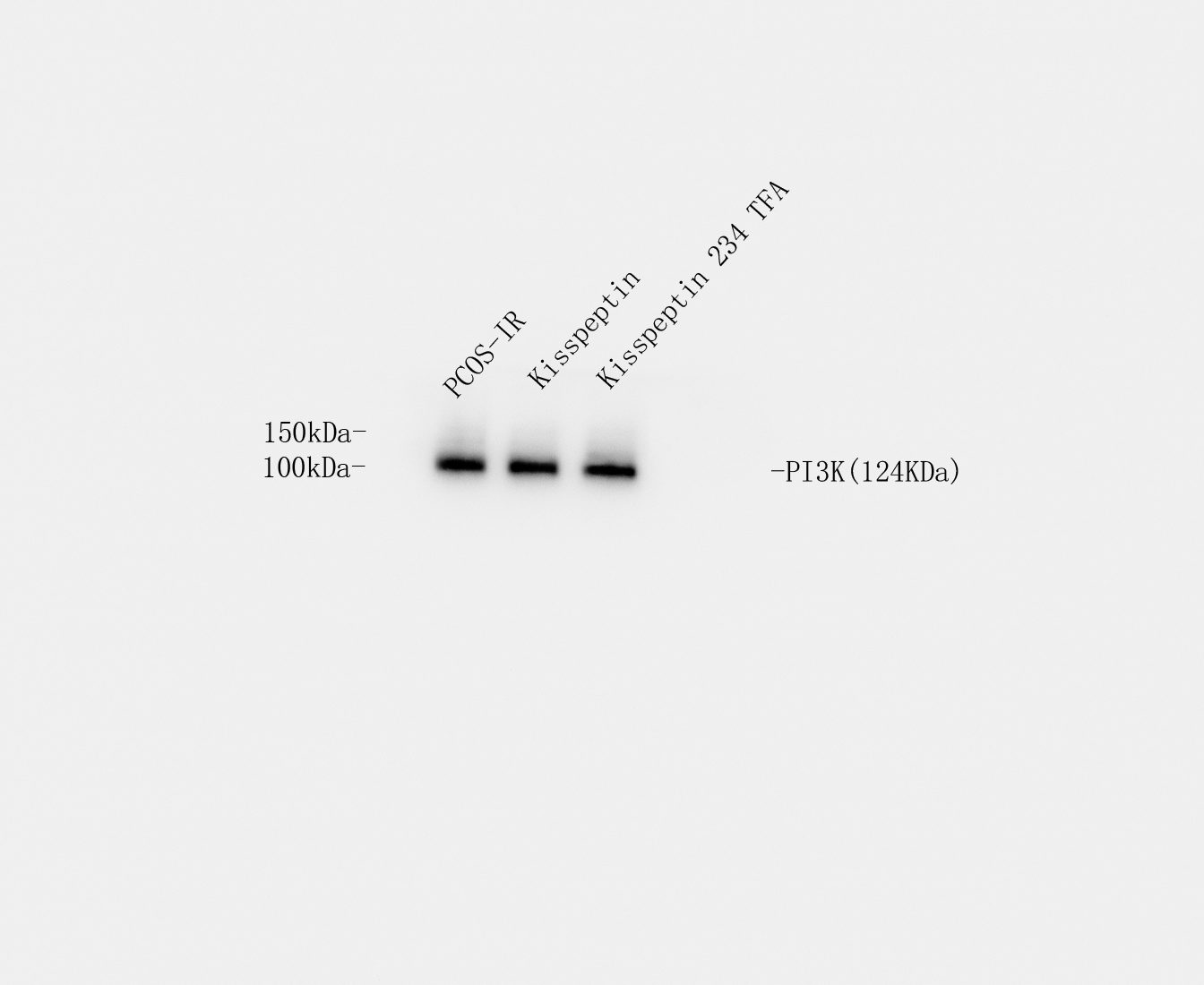

Supplement: S1 Data — (ZIP) [file pone.0342158.s001.zip › Supporting Information files/Raw data/FIG3/labeled blot image-PI3K(1).tif]

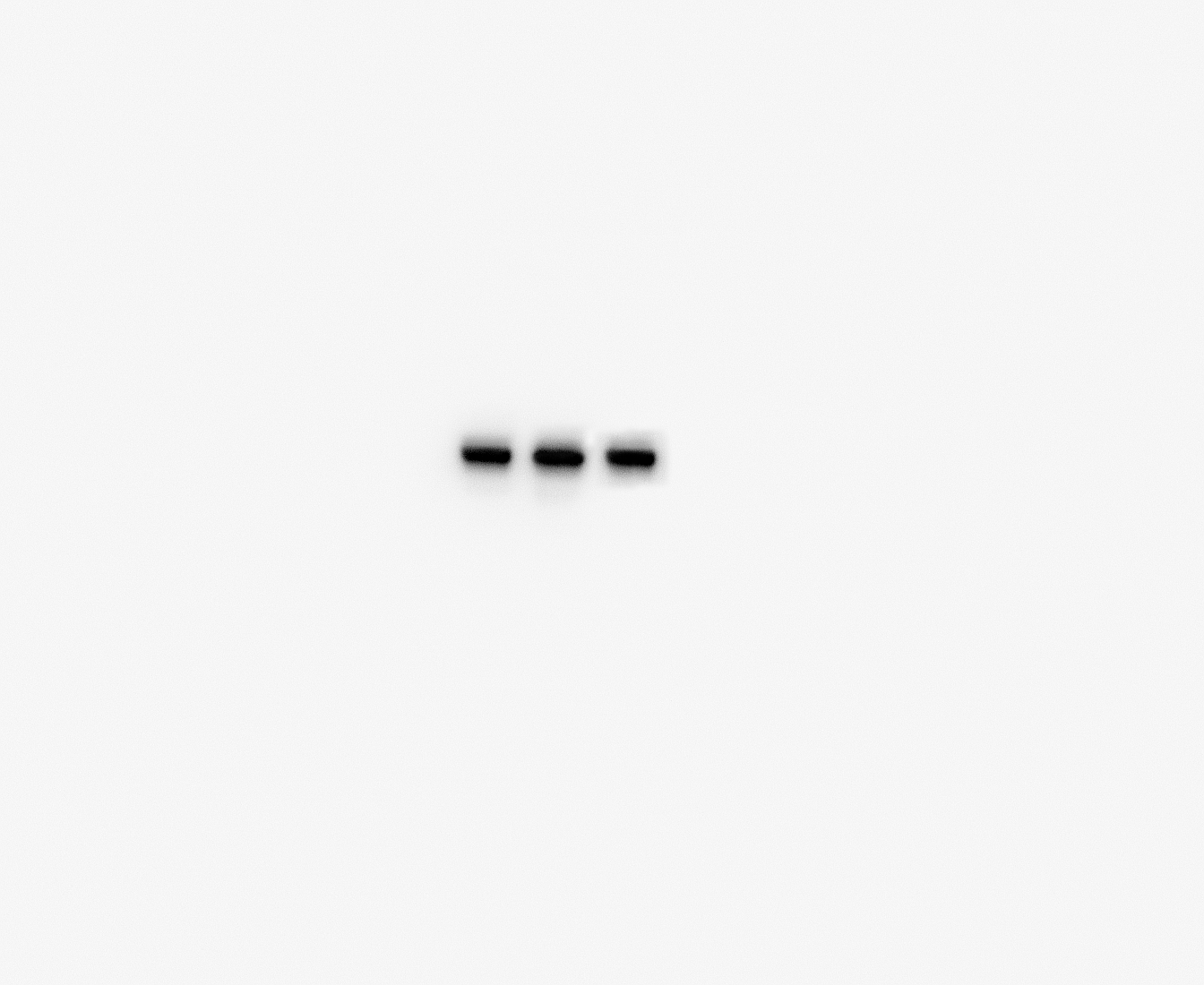

Supplement: S1 Data — (ZIP) [file pone.0342158.s001.zip › Supporting Information files/Raw data/FIG3/original blot imagge-actin.tif]

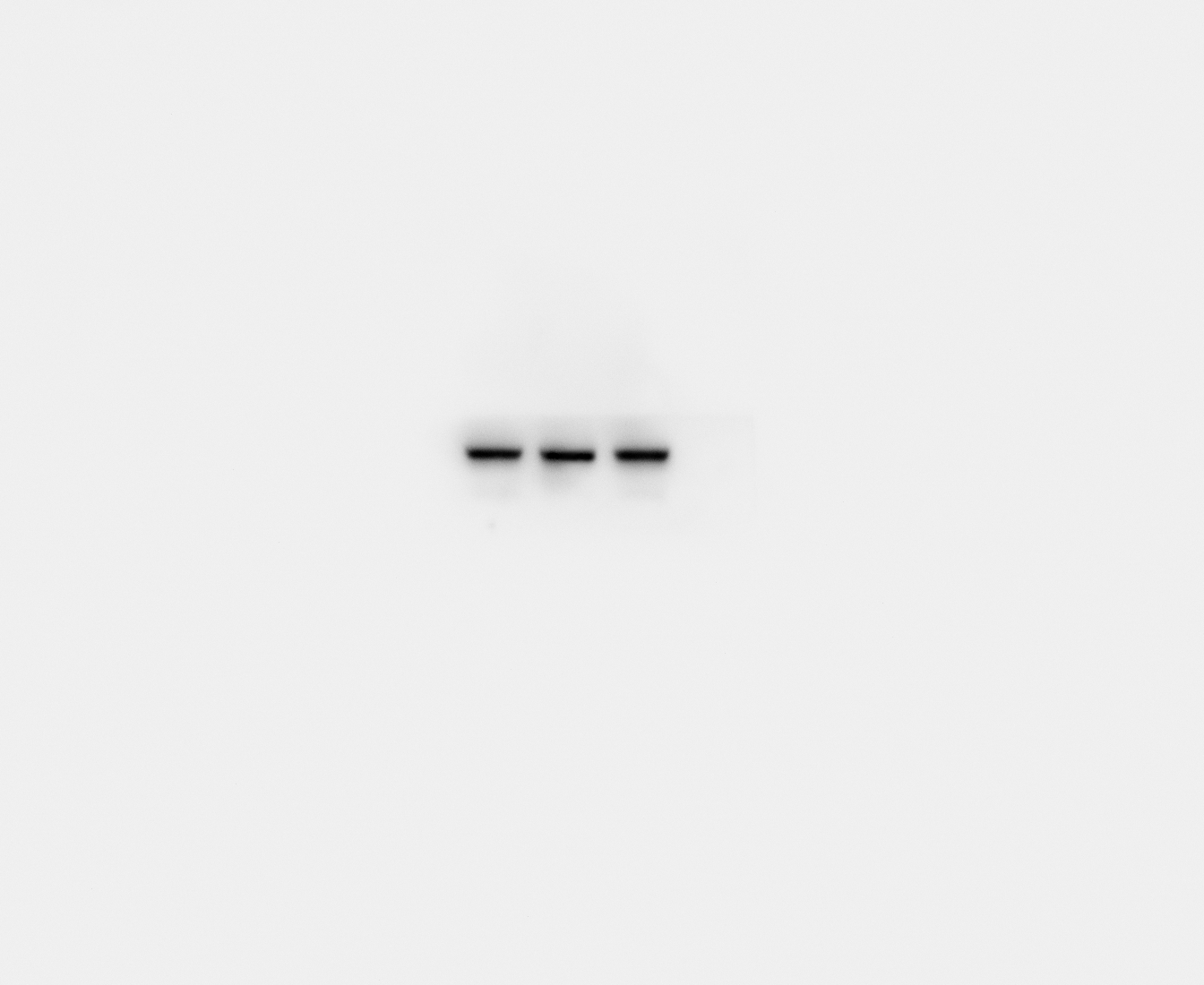

Supplement: S1 Data — (ZIP) [file pone.0342158.s001.zip › Supporting Information files/Raw data/FIG3/original blot imagge-AKT.Tif]

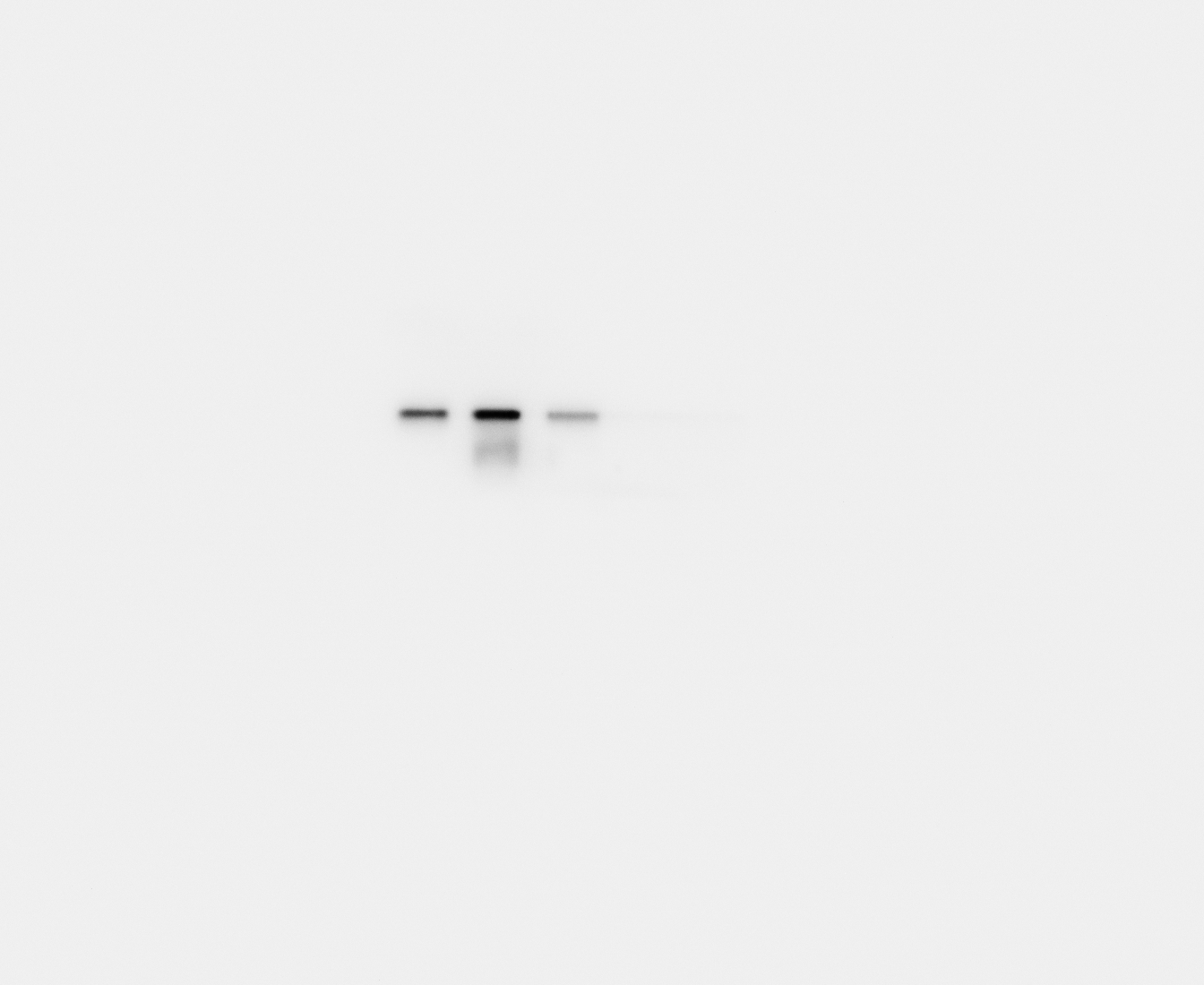

Supplement: S1 Data — (ZIP) [file pone.0342158.s001.zip › Supporting Information files/Raw data/FIG3/original blot imagge-GLUT4.Tif]

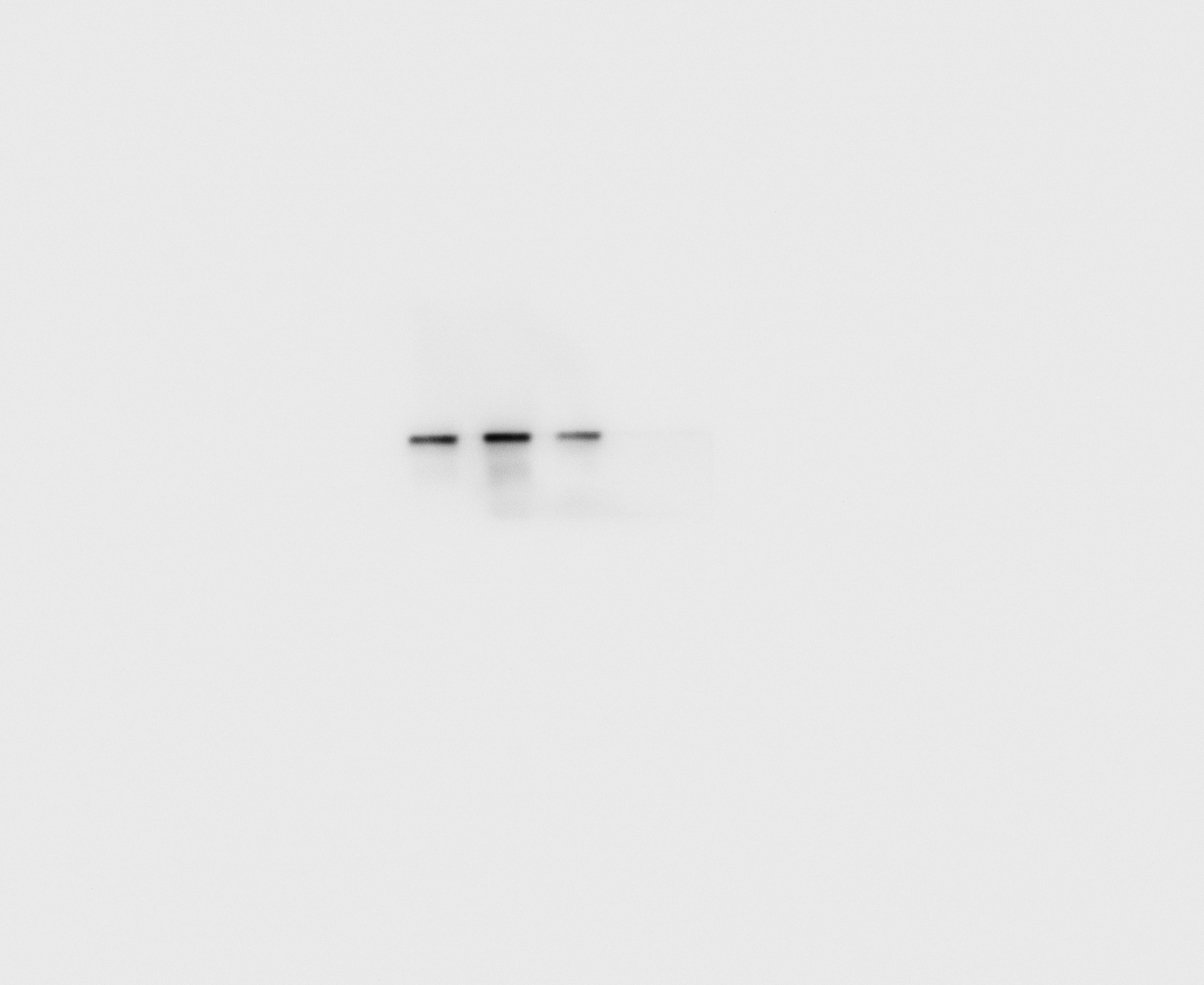

Supplement: S1 Data — (ZIP) [file pone.0342158.s001.zip › Supporting Information files/Raw data/FIG3/original blot imagge-p-AKT.Tif]

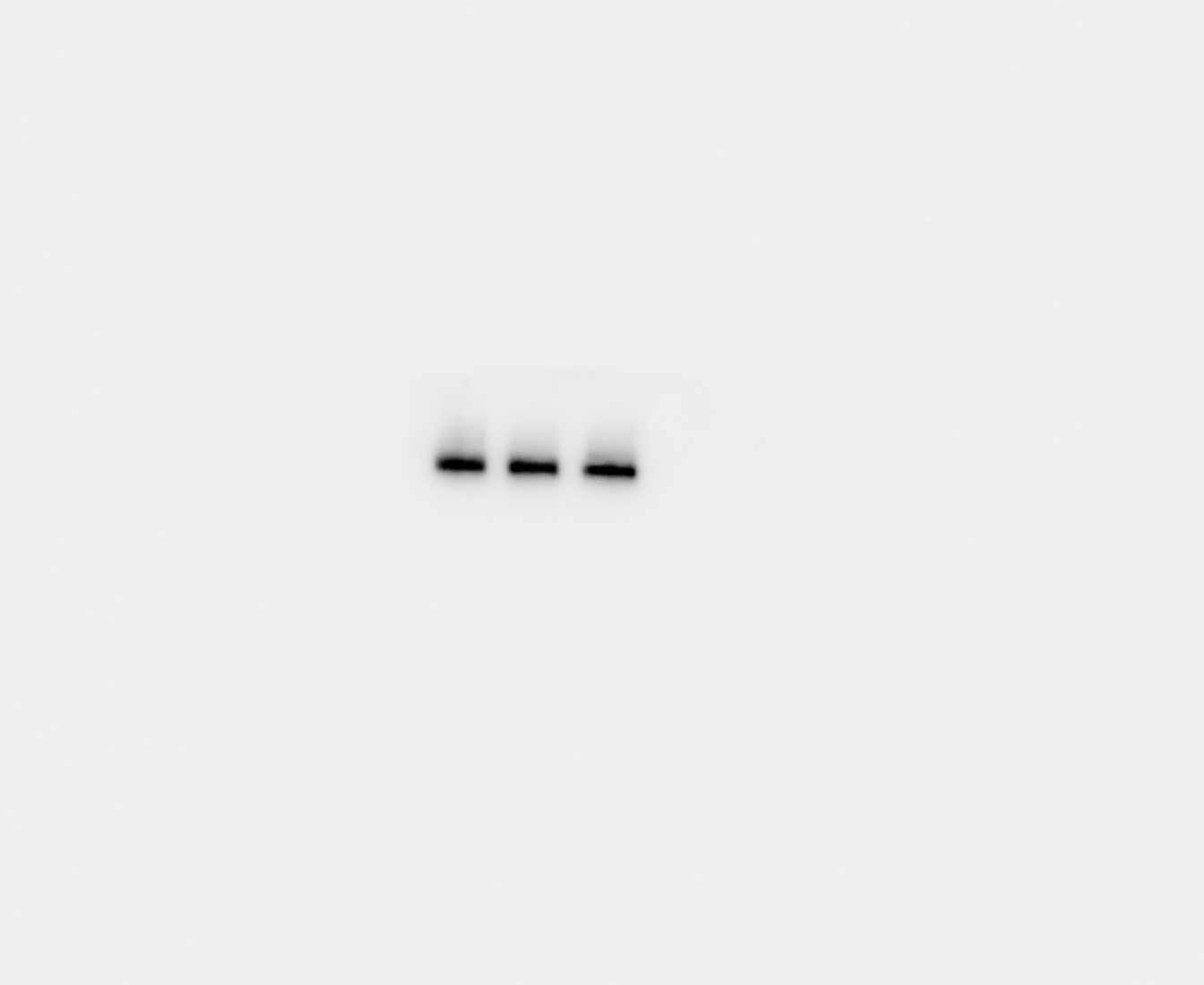

Supplement: S1 Data — (ZIP) [file pone.0342158.s001.zip › Supporting Information files/Raw data/FIG3/original blot imagge-PI3K.Tif]

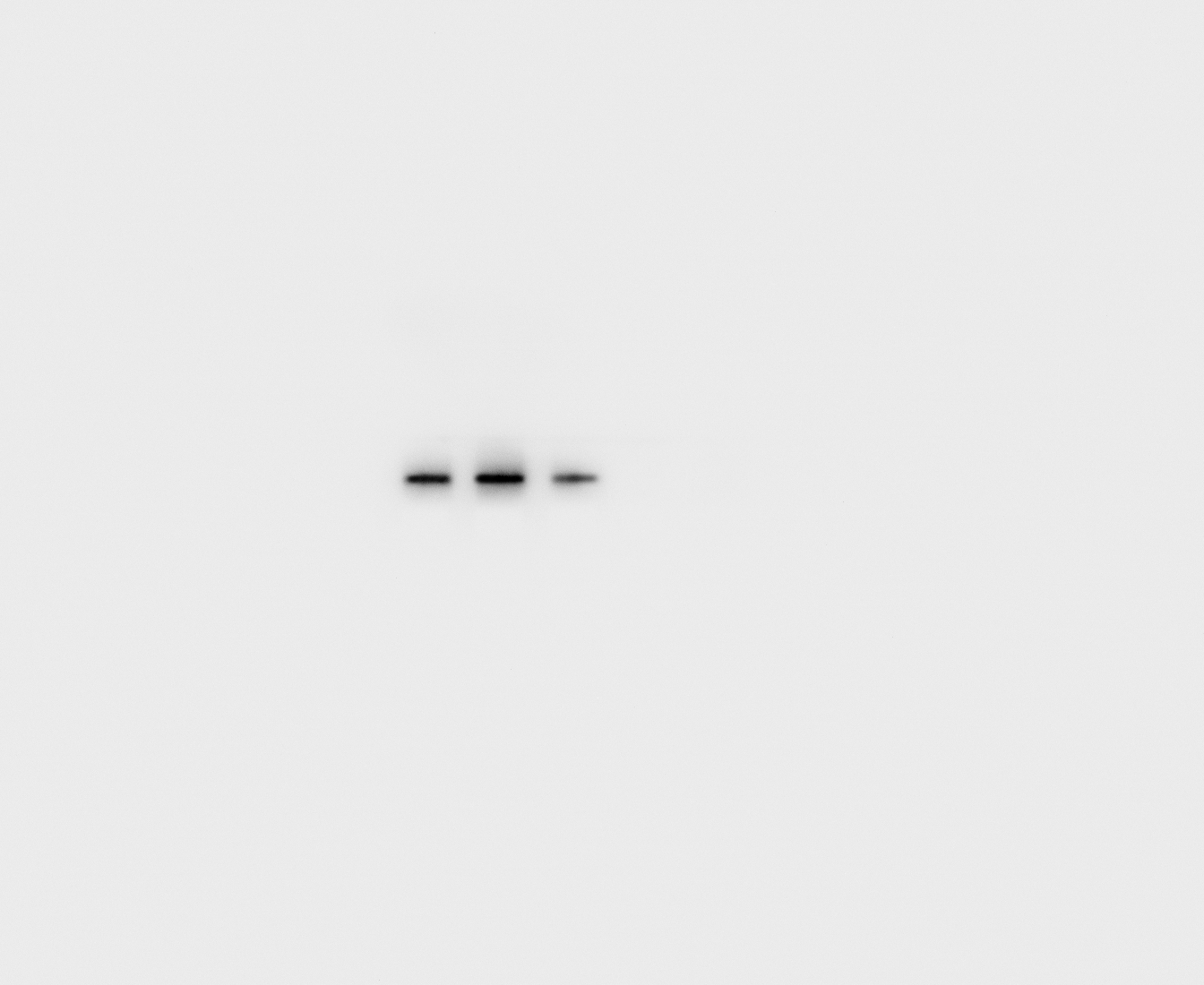

Supplement: S1 Data — (ZIP) [file pone.0342158.s001.zip › Supporting Information files/Raw data/FIG3/original blot-imagge-P-PI3K.Tif]

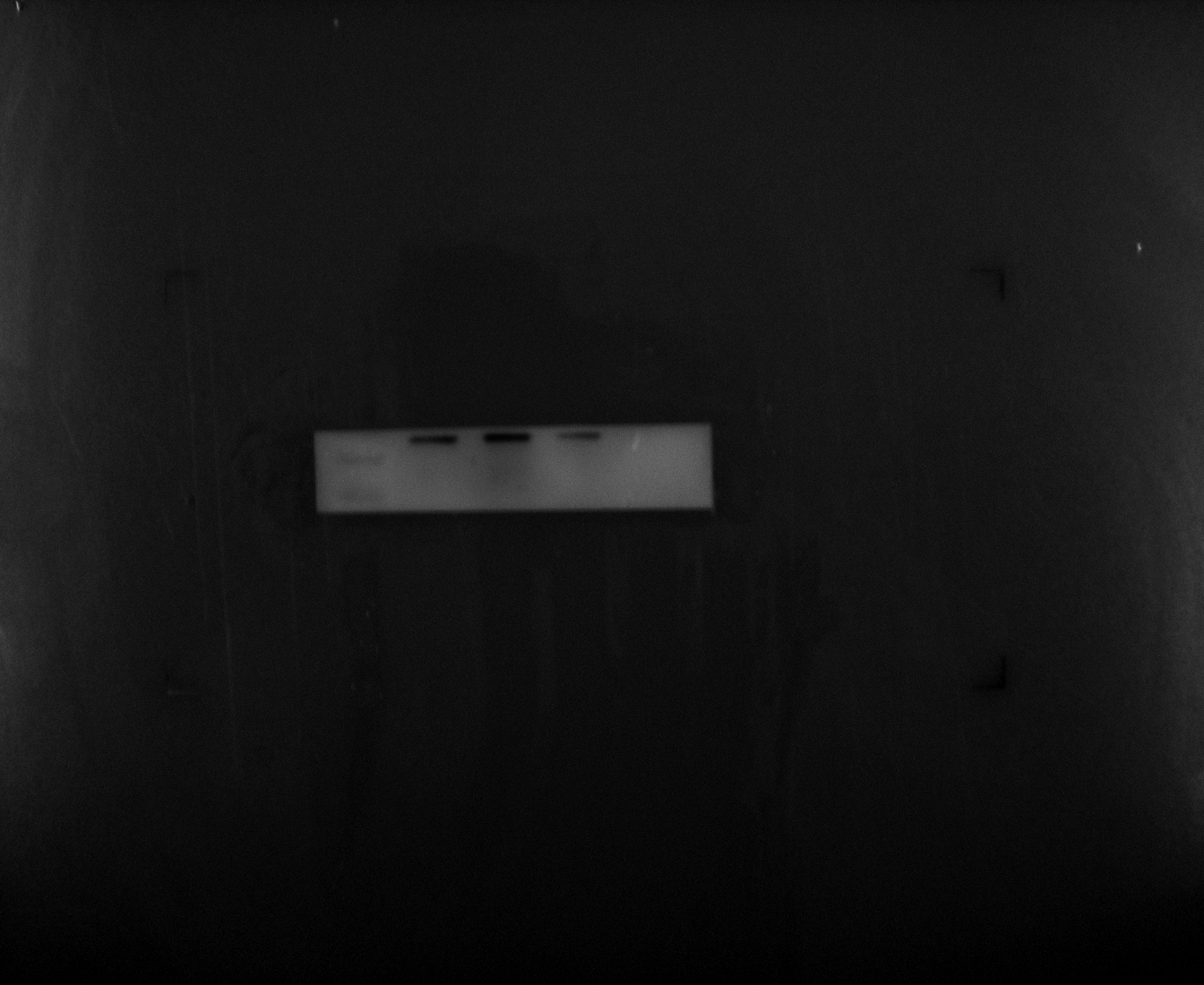

Supplement: S1 Data — (ZIP) [file pone.0342158.s001.zip › Supporting Information files/Raw data/FIG3/p-AKT merge.tif]

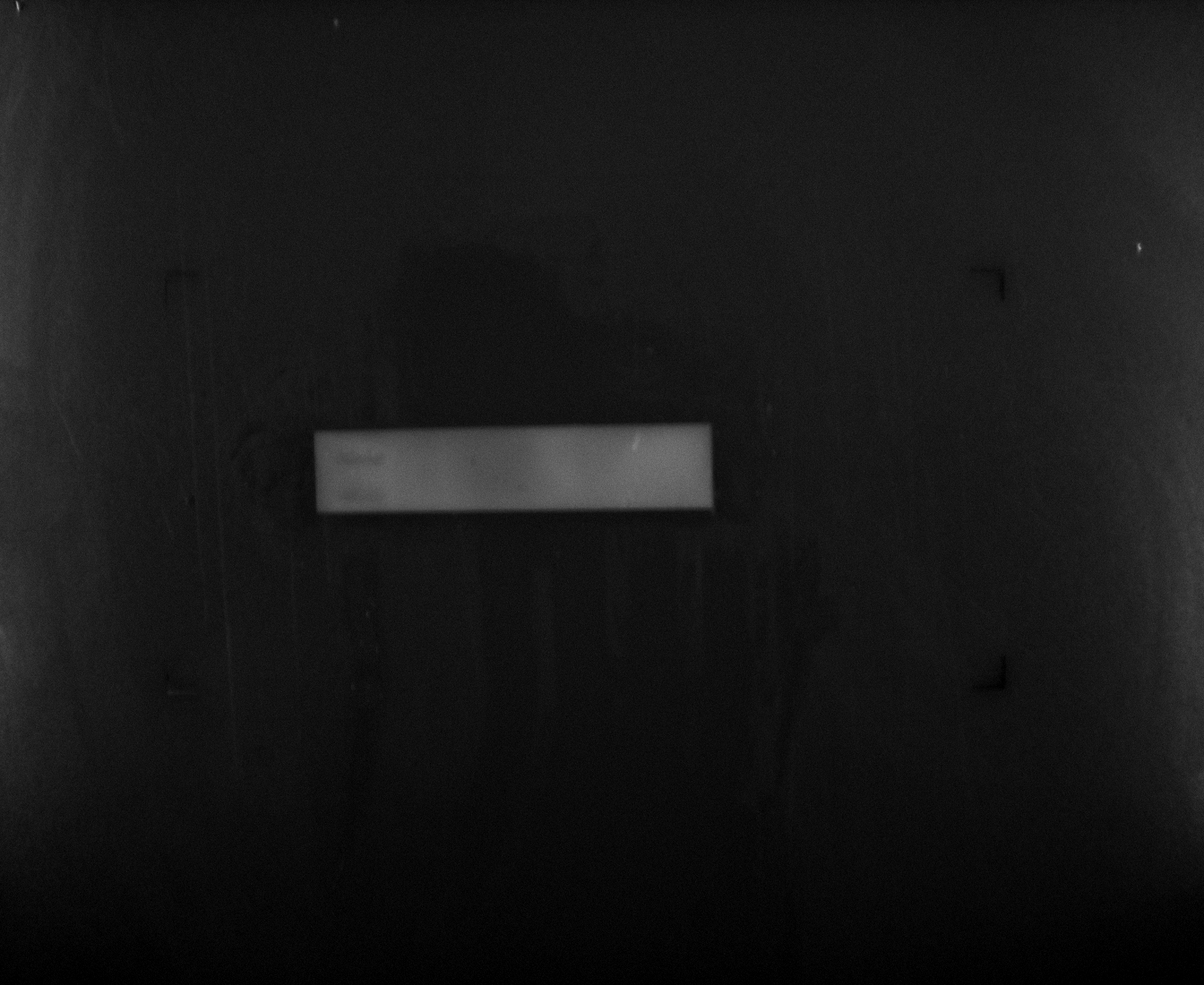

Supplement: S1 Data — (ZIP) [file pone.0342158.s001.zip › Supporting Information files/Raw data/FIG3/p-AKT white light.Tif]

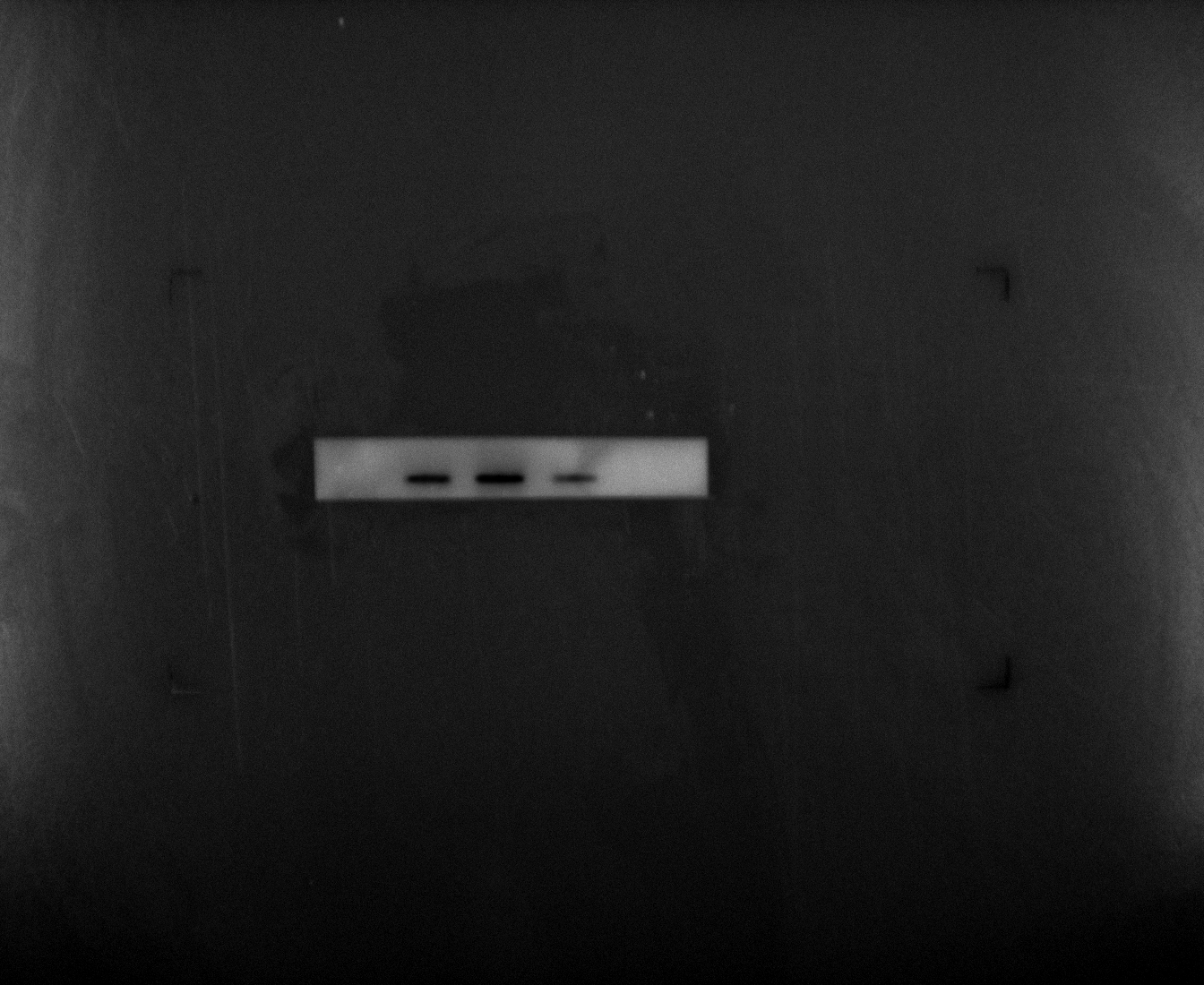

Supplement: S1 Data — (ZIP) [file pone.0342158.s001.zip › Supporting Information files/Raw data/FIG3/P-PI3K merge.tif]

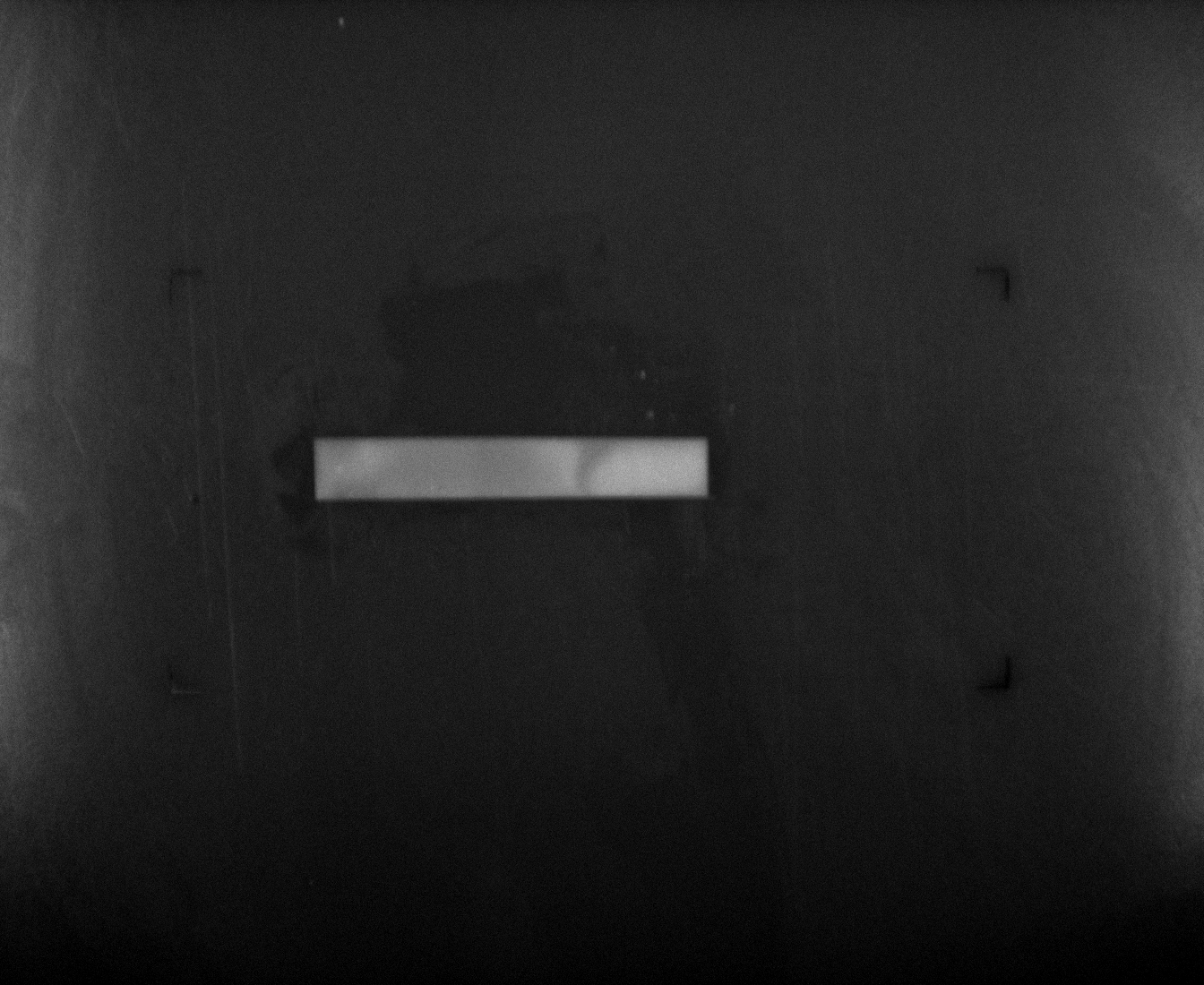

Supplement: S1 Data — (ZIP) [file pone.0342158.s001.zip › Supporting Information files/Raw data/FIG3/P-PI3K white light.Tif]

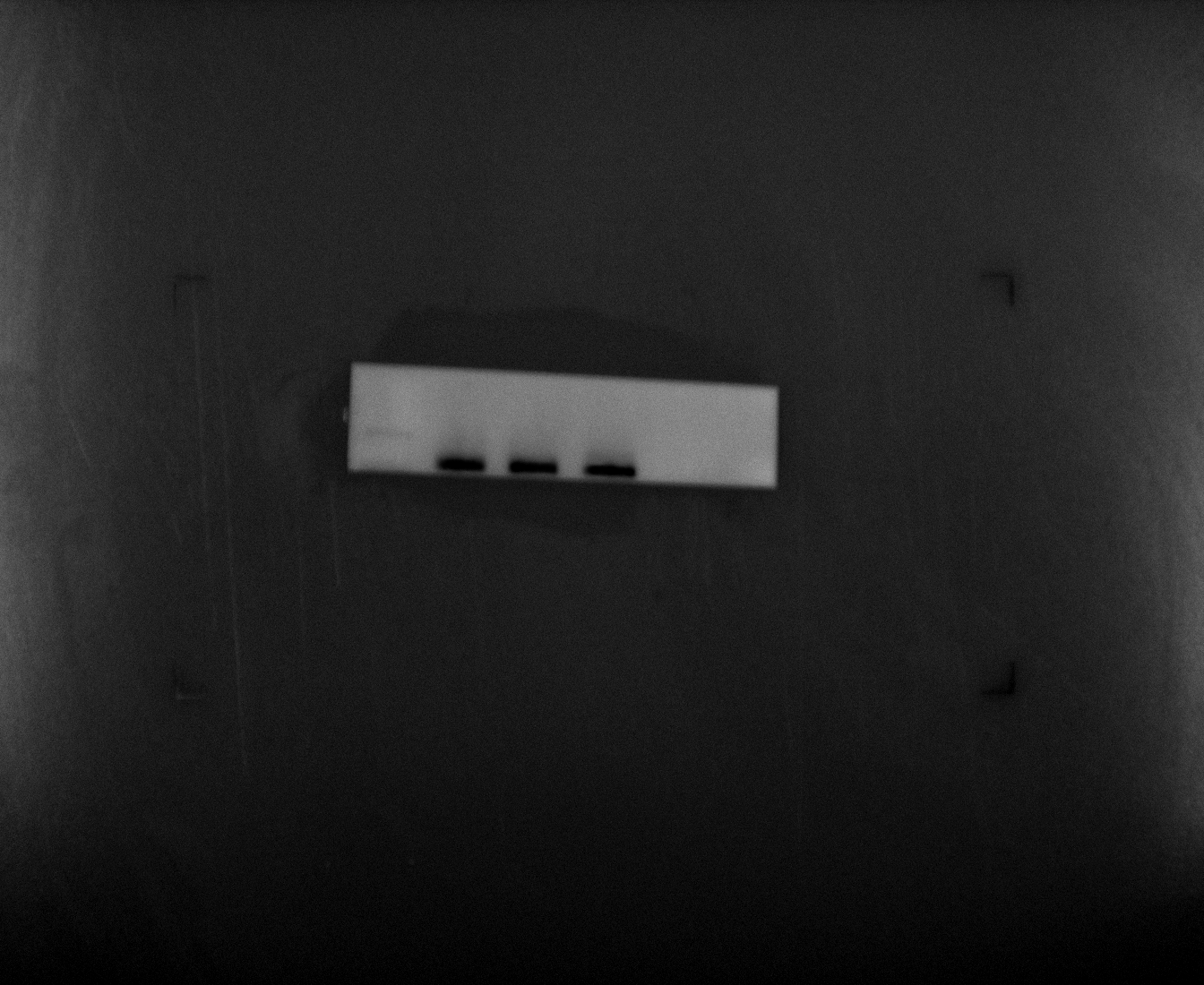

Supplement: S1 Data — (ZIP) [file pone.0342158.s001.zip › Supporting Information files/Raw data/FIG3/PI3K merge.tif]

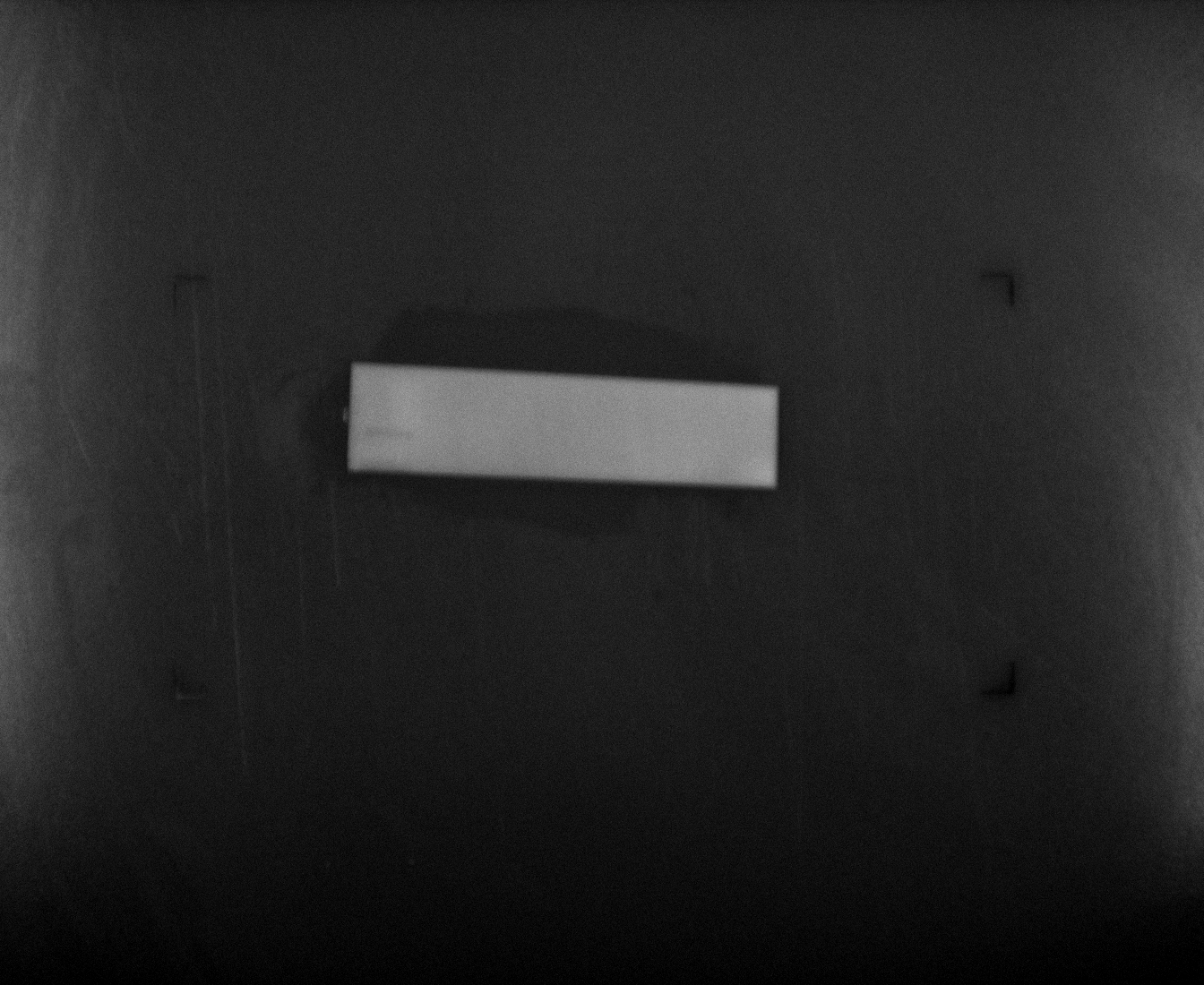

Supplement: S1 Data — (ZIP) [file pone.0342158.s001.zip › Supporting Information files/Raw data/FIG3/PI3K white light.Tif]

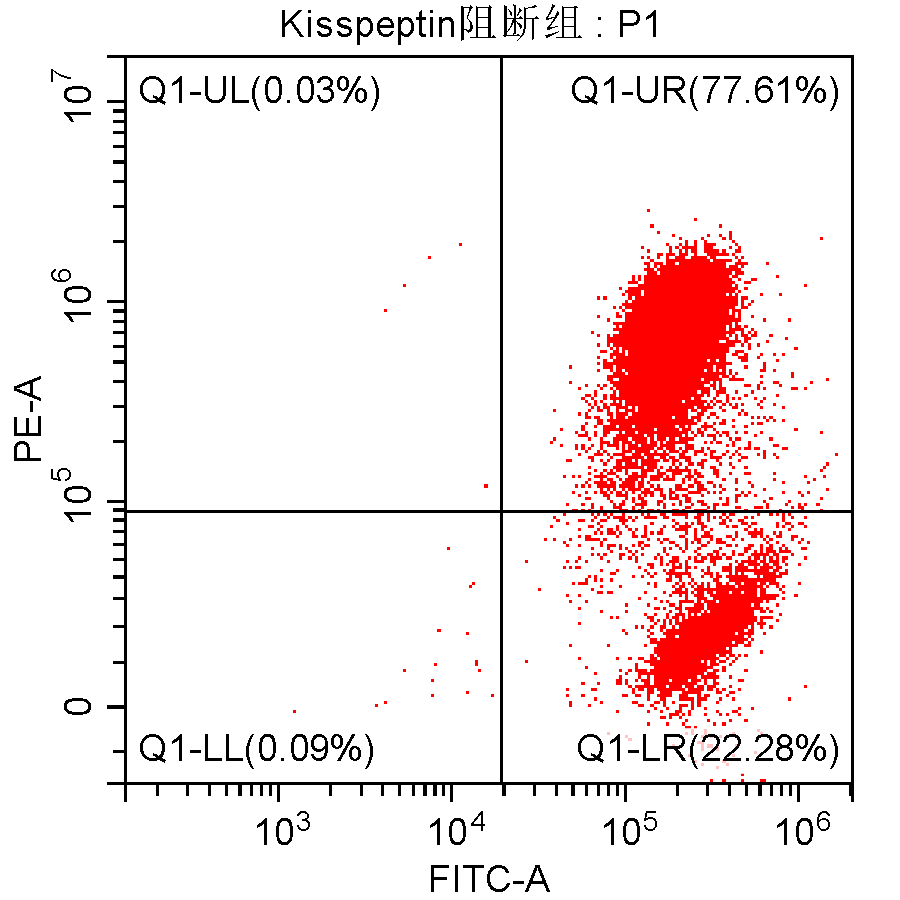

Supplement: S1 Data — (ZIP) [file pone.0342158.s001.zip › Supporting Information files/Raw data/FIG4/Kisspeptin 234 TFA_MMP.bmp]

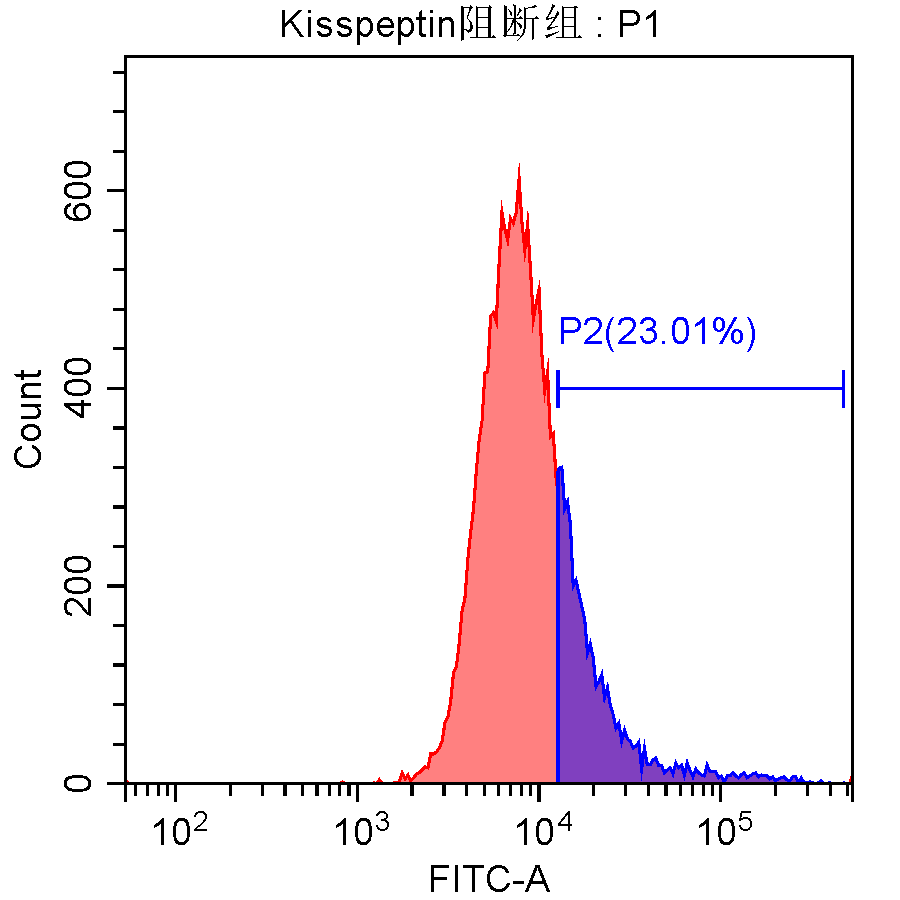

Supplement: S1 Data — (ZIP) [file pone.0342158.s001.zip › Supporting Information files/Raw data/FIG4/Kisspeptin 234 TFA_ROS.bmp]

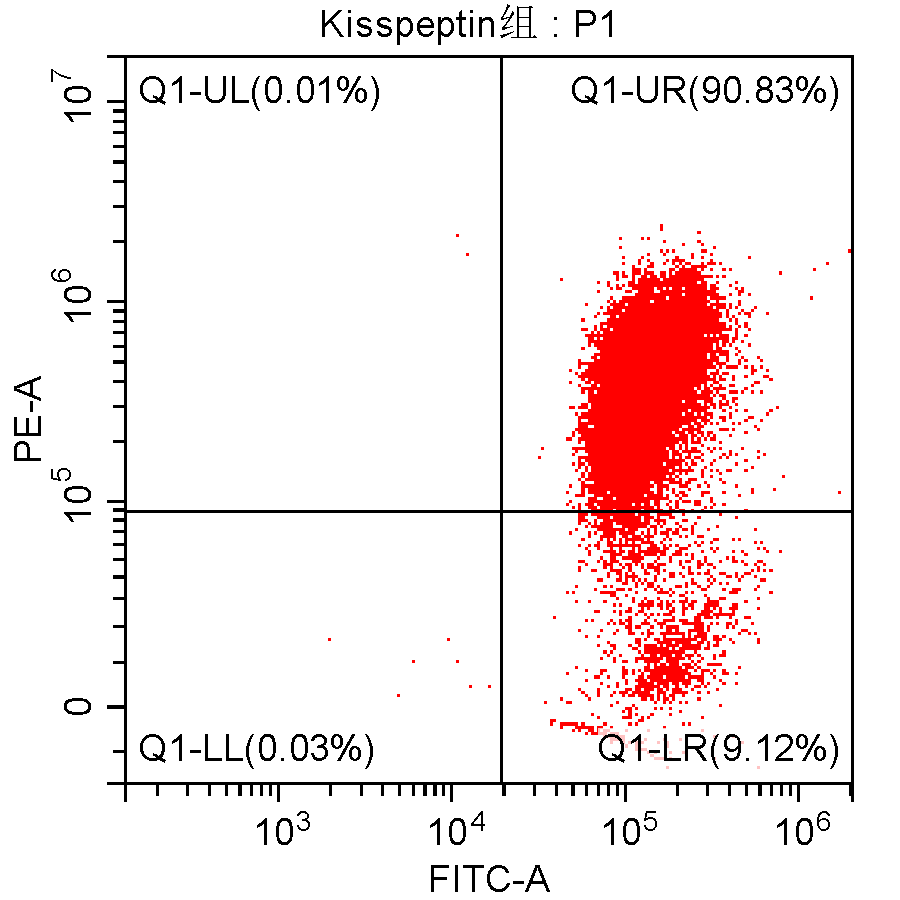

Supplement: S1 Data — (ZIP) [file pone.0342158.s001.zip › Supporting Information files/Raw data/FIG4/Kisspeptin_MMP.bmp]

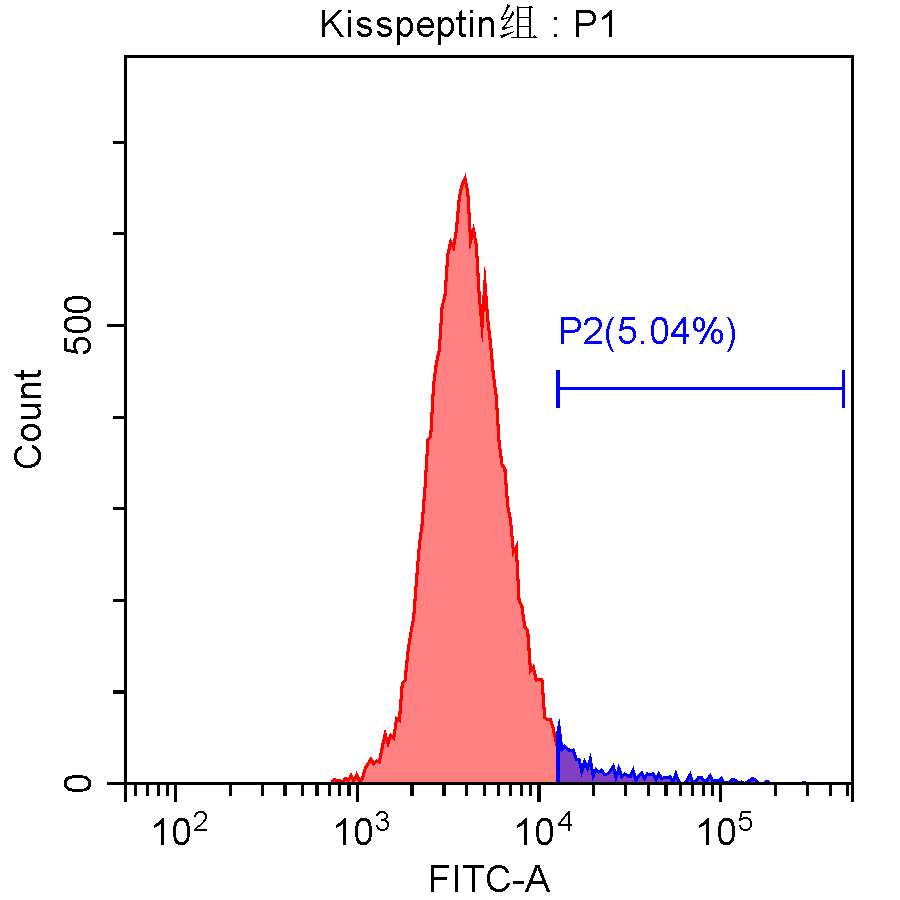

Supplement: S1 Data — (ZIP) [file pone.0342158.s001.zip › Supporting Information files/Raw data/FIG4/Kisspeptin_ROS.bmp]

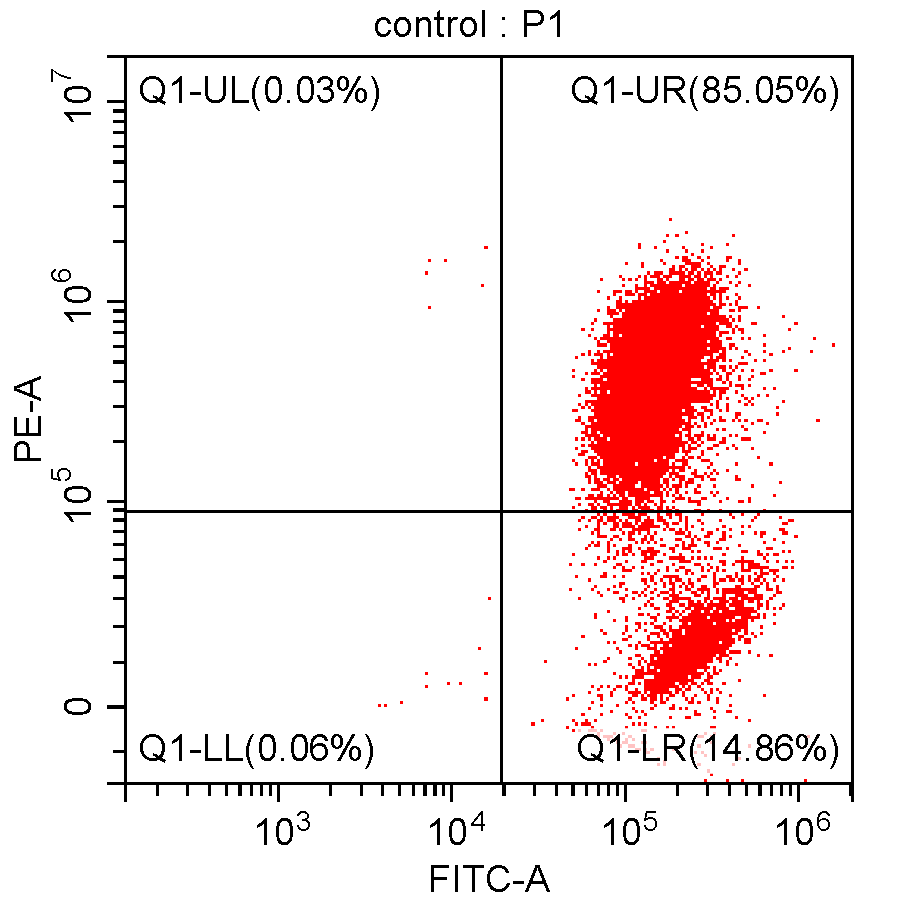

Supplement: S1 Data — (ZIP) [file pone.0342158.s001.zip › Supporting Information files/Raw data/FIG4/PCOS-IR_MMP.bmp]

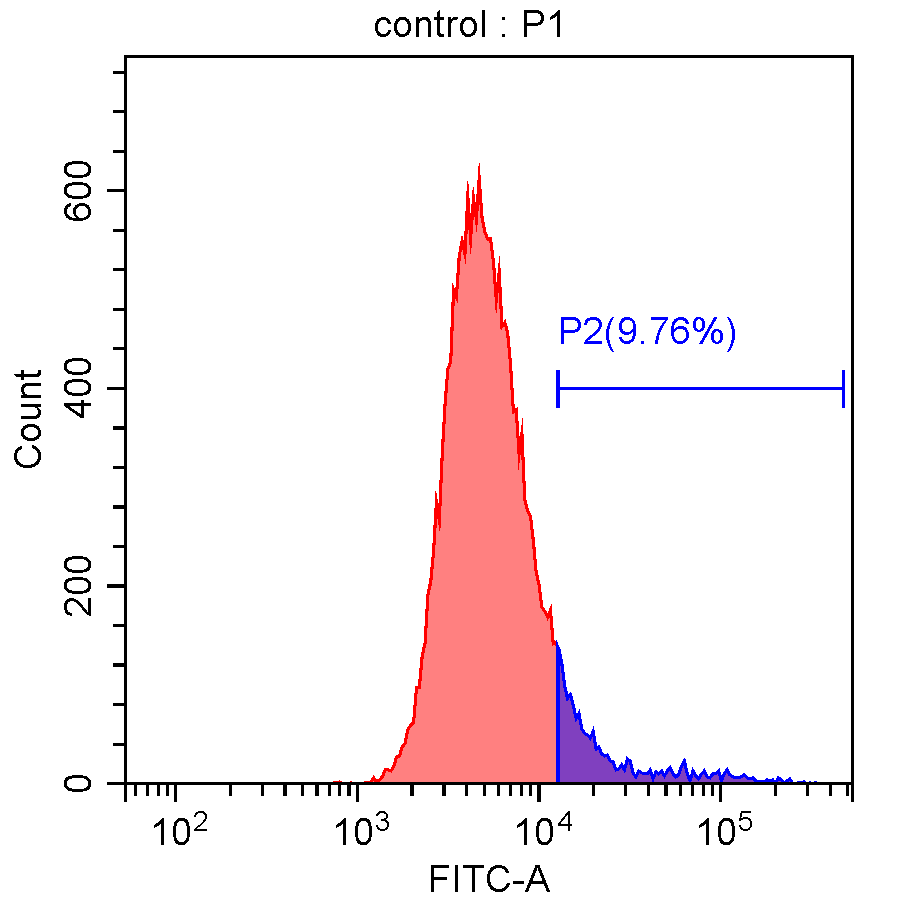

Supplement: S1 Data — (ZIP) [file pone.0342158.s001.zip › Supporting Information files/Raw data/FIG4/PCOS-IR_ROS.bmp]
