# Supplementary material for: Kisspeptin improves local ovarian insulin resistance in PCOS by modulating the PI3K/AKT/GLUT4 signaling pathway
Source: PLoS One. 2026 Feb 2;21(2):e0342158. doi: 10.1371/journal.pone.0342158 (PMC12863573; doi:10.1371/journal.pone.0342158)
Supplement: Attachment — Submitted filename: Response to Reviewers.docx [file pone.0342158.s005.docx]

We've checked your submission and before we can proceed, we need you to address the following issues:

1.  PLOS ONE now requires that authors provide the original uncropped and unadjusted images underlying all blot or gel results reported in a submission’s figures or Supporting Information files. This policy and the journal’s other requirements for blot/gel reporting and figure preparation are described in detail at https://journals.plos.org/plosone/s/figures#loc-blot-and-gel-reporting-requirements and https://journals.plos.org/plosone/s/figures#loc-preparing-figures-from-image-files. When you submit your revised manuscript, please ensure that your figures adhere fully to these guidelines and provide the original underlying images for all blot or gel data reported in your submission. See the following link for instructions on providing the original image data: https://journals.plos.org/plosone/s/figures#loc-original-images-for-blots-and-gels.
In your cover letter, please note whether your blot/gel image data are in Supporting Information or posted at a public data repository, provide the repository URL if relevant, and provide specific details as to which raw blot/gel images, if any, are not available. Email us at plosone@plos.org if you have any questions.

Answer: Thank you for your feedback. We have submitted all the original images of the blot or gel results as instructed and have made the formatting corrections and file naming strictly according to your requirements. Please kindly review the latest files we have provided.

2. "We note that your Data Availability Statement is currently as follows: [All relevant data are within the manuscript and its Supporting Information files.]

Please confirm at this time whether or not your submission contains all raw data required to replicate the results of your study. Authors must share the “minimal data set” for their submission. PLOS defines the minimal data set to consist of the data required to replicate all study findings reported in the article, as well as related metadata and methods (https://journals.plos.org/plosone/s/data-availability#loc-minimal-data-set-definition).

For example, authors should submit the following data:
- The values behind the means, standard deviations and other measures reported;
- The values used to build graphs;
- The points extracted from images for analysis.

Authors do not need to submit their entire data set if only a portion of the data was used in the reported study.

If your submission does not contain these data, please either upload them as Supporting Information files or deposit them to a stable, public repository and provide us with the relevant URLs, DOIs, or accession numbers. For a list of recommended repositories, please see https://journals.plos.org/plosone/s/recommended-repositories.

If there are ethical or legal restrictions on sharing a de-identified data set, please explain them in detail (e.g., data contain potentially sensitive information, data are owned by a third-party organization, etc.) and who has imposed them (e.g., an ethics committee). Please also provide contact information for a data access committee, ethics committee, or other institutional body to which data requests may be sent. If data are owned by a third party, please indicate how others may request data access."

Answer: Thank you for your kind reminder once again. We confirm that our submission includes all the original data (minimal data set) required to replicate the results of this study.


3. Please upload a Response to Reviewers letter which should include a point by point response to each of the points made by the Editor and / or Reviewers.

(This should be uploaded as a 'Response to Reviewers' file type.)

Please follow this link for more information: http://blogs.PLOS.org/everyone/2011/05/10/how-to-submit-your-revised-manuscript/

Answer: Thank you for your reminder. We have provided the Response to Reviewers letter as instructed. Please kindly check the latest file we have submitted.

4. Please ensure that you include the author list within your main document and ensure that each author is linked to an affiliation.

Answer: Thank you again for your feedback. We have added the author information to the main document as instructed. Please kindly review the latest version of the main document we have provided.
