# Supplementary material for: Kisspeptin improves local ovarian insulin resistance in PCOS by modulating the PI3K/AKT/GLUT4 signaling pathway
Source: PLoS One. 2026 Feb 2;21(2):e0342158. doi: 10.1371/journal.pone.0342158 (PMC12863573; doi:10.1371/journal.pone.0342158)
Supplement: Attachment — Submitted filename: 2 nd review.docx [file pone.0342158.s004.docx]

the authors have addressed most questions and added supplementary data on testosterone and insulin which is highly appreciated. However, some issues need to be addressed.

1. Fig 1A- There are no arrow marks. Please add them.
2. Please indicate changes using coloring in the text.
3. Reviewer question: Please indicate the line numbers for the addition regarding kisspeptin receptor expression in granulosa cells. I can’t find them.

“Answer: We thank the reviewer for this insightful comment. In response, we have clarified in the Discussion section that the rationale for our study is based on local ovarian production of kisspeptin, rather than circulating serum levels. Although kisspeptin was added to the culture media in vitro to mimic its physiological effects, the in vivo relevance is primarily due to its local synthesis within the ovary. “Furthermore, we have now included information regarding kisspeptin receptor (KISS1R) expression in granulosa cells, which was measured and confirmed, in the revised manuscript”.
