# Supplementary material for: Kisspeptin improves local ovarian insulin resistance in PCOS by modulating the PI3K/AKT/GLUT4 signaling pathway
Source: PLoS One. 2026 Feb 2;21(2):e0342158. doi: 10.1371/journal.pone.0342158 (PMC12863573; doi:10.1371/journal.pone.0342158)
Supplement: Attachment — Submitted filename: Response_to_Reviewers_auresp_2.docx [file pone.0342158.s006.docx]

the authors have addressed most questions and added supplementary data on testosterone and insulin which is highly appreciated. However, some issues need to be addressed.

1. Fig 1A- There are no arrow marks. Please add them.

Answer: Thank you very much for your valuable suggestion. We sincerely apologize for the confusion caused by our oversight. In accordance with your instructions, we have replaced Figure 1, and we kindly invite you to review the updated version now provided in the revised manuscript.

2. Please indicate changes using coloring in the text.

Answer: Thank you for your feedback. We have highlighted all revised sections in the manuscript as instructed. Please review the latest version we have provided.

3. Reviewer question: Please indicate the line numbers for the addition regarding kisspeptin receptor expression in granulosa cells. I can’t find them.

“Answer: We thank the reviewer for this insightful comment. In response, we have clarified in the Discussion section that the rationale for our study is based on local ovarian production of kisspeptin, rather than circulating serum levels. Although kisspeptin was added to the culture media in vitro to mimic its physiological effects, the in vivo relevance is primarily due to its local synthesis within the ovary. “Furthermore, we have now included information regarding kisspeptin receptor (KISS1R) expression in granulosa cells, which was measured and confirmed, in the revised manuscript”.

Answer: We sincerely thank the reviewer for carefully checking our previous revision. We apologize for the oversight—although we stated in the last response that information regarding KISS1R expression in granulosa cells had been added, the corresponding text was not clearly incorporated in the revised manuscript, which caused confusion. We appreciate the reviewer’s patience in pointing this out.

In the current revision, we have corrected this issue and have now explicitly added the relevant description based on published evidence. The revised manuscript now includes the following statement in the Discussion section:

“In addition, previous studies have shown that kisspeptin is specifically expressed in granulosa cells, whereas its receptor KISS1R exhibits specific expression in oocytes.”

This information clarifies the cell-specific distribution of kisspeptin and its receptor in the ovary, thereby strengthening the mechanistic rationale of our study.

We appreciate the reviewer’s valuable comments, which have helped us improve the accuracy and clarity of the manuscript.
