# Supplementary material for: Kisspeptin improves local ovarian insulin resistance in PCOS by modulating the PI3K/AKT/GLUT4 signaling pathway
Source: PLoS One. 2026 Feb 2;21(2):e0342158. doi: 10.1371/journal.pone.0342158 (PMC12863573; doi:10.1371/journal.pone.0342158)
Supplement: Attachment — Submitted filename: Response_to_Reviewers_auresp_3.docx [file pone.0342158.s007.docx]

Dear Dr. Ma,

Thank you for submitting your manuscript to PLOS ONE. After careful consideration, we feel that it has merit but does not fully meet PLOS ONE’s publication criteria as it currently stands. Therefore, we invite you to submit a revised version of the manuscript that addresses the points raised during the review process.

=====================
Request from the Editorial Office: We noted that the following related study was recently published by some of your coauthors:

Kisspeptin regulates the proliferation and apoptosis of ovary granulosa cells in polycystic ovary syndrome by modulating the PI3K/AKT/ERK signalling pathway (https://doi.org/10.1186/s12905-022-02154-6)

In order to comply with our Publication Criteria (http://journals.plos.org/plosone/s/criteria-for-publication#loc-2), please discuss this study in your Introduction and provide a sound scientific rationale for the submitted work. In your Discussion section, please discuss your findings in light of this existing paper. Please contact [plosone@plos.org](mailto:plosone@plos.org) with any questions about this request. Thank you.
=====================

Response: Thank you very much for your valuable suggestions. We have revised and expanded the Introduction and Discussion sections in accordance with your comments. Please kindly review the latest version of our manuscript.

Please submit your revised manuscript by Feb 05 2026 11:59PM. If you will need more time than this to complete your revisions, please reply to this message or contact the journal office at [plosone@plos.org](mailto:plosone@plos.org). When you're ready to submit your revision, log on to https://www.editorialmanager.com/pone/ and select the 'Submissions Needing Revision' folder to locate your manuscript file.

Please include the following items when submitting your revised manuscript:

- A letter that responds to each point raised by the academic editor and reviewer(s). You should upload this letter as a separate file labeled 'Response to Reviewers'.
- A marked-up copy of your manuscript that highlights changes made to the original version. You should upload this as a separate file labeled 'Revised Manuscript with Track Changes'.
- An unmarked version of your revised paper without tracked changes. You should upload this as a separate file labeled 'Manuscript'.

If you would like to make changes to your financial disclosure, please include your updated statement in your cover letter. Guidelines for resubmitting your figure files are available below the reviewer comments at the end of this letter.

If applicable, we recommend that you deposit your laboratory protocols in protocols.io to enhance the reproducibility of your results. Protocols.io assigns your protocol its own identifier (DOI) so that it can be cited independently in the future. For instructions see: [https://journals.plos.org/plosone/s/submission-guidelines#loc-laboratory-protocols](https://track.editorialmanager.com/CL0/https://journals.plos.org/plosone/s/submission-guidelines%23loc-laboratory-protocols/1/010f019b4dda96e4-430cd5f9-38f3-4573-ab26-6aa2f804619a-000000/0irtAGuqq3rLDA3BJCcTytG57jo0LtVaKq7R6Ho-r44=243). Additionally, PLOS ONE offers an option for publishing peer-reviewed Lab Protocol articles, which describe protocols hosted on protocols.io. Read more information on sharing protocols at [https://plos.org/protocols?utm_medium=editorial-email&utm_source=authorletters&utm_campaign=protocols](https://track.editorialmanager.com/CL0/https://plos.org/protocols?utm_medium=editorial-email&utm_source=authorletters&utm_campaign=protocols/1/010f019b4dda96e4-430cd5f9-38f3-4573-ab26-6aa2f804619a-000000/7p36cF7axLhaLdRY1lQszpOvAByTjWSODTt6Xt4Hcmg=243).

We look forward to receiving your revised manuscript.

Kind regards,

Sarah Jose, Ph.D.
Staff Editor
PLOS One

Journal Requirements:

1. If the reviewer comments include a recommendation to cite specific previously published works, please review and evaluate these publications to determine whether they are relevant and should be cited. There is no requirement to cite these works unless the editor has indicated otherwise.
   Response: Thank you very much for your reminder.

2. Please review your reference list to ensure that it is complete and correct. If you have cited papers that have been retracted, please include the rationale for doing so in the manuscript text, or remove these references and replace them with relevant current references. Any changes to the reference list should be mentioned in the rebuttal letter that accompanies your revised manuscript. If you need to cite a retracted article, indicate the article’s retracted status in the References list and also include a citation and full reference for the retraction notice.
Response: Thank you very much for your reminder.


[Note: HTML markup is below. Please do not edit.]

[NOTE: If reviewer comments were submitted as an attachment file, they will be attached to this email and accessible via the submission site. Please log into your account, locate the manuscript record, and check for the action link "View Attachments". If this link does not appear, there are no attachment files.]

To ensure your figures meet our technical requirements, please review our figure guidelines: https://journals.plos.org/plosone/s/figures

You may also use PLOS’s free figure tool, NAAS, to help you prepare publication quality figures: https://journals.plos.org/plosone/s/figures#loc-tools-for-figure-preparation.

NAAS will assess whether your figures meet our technical requirements by comparing each figure against our figure specifications.
