# Supplementary material for: Kisspeptin improves local ovarian insulin resistance in PCOS by modulating the PI3K/AKT/GLUT4 signaling pathway
Source: PLoS One. 2026 Feb 2;21(2):e0342158. doi: 10.1371/journal.pone.0342158 (PMC12863573; doi:10.1371/journal.pone.0342158)
Supplement: Attachment — Submitted filename: Response_to_Reviewers_auresp_4.docx [file pone.0342158.s008.docx]

==============================
**ACADEMIC EDITOR:**

Please specify the major differences between your article and the newly published study [Kisspeptin regulates the proliferation and apoptosis of ovary granulosa cells in polycystic ovary syndrome by modulating the PI3K/AKT/ERK signalling pathway (https://doi.org/10.1186/s12905-022-02154-6)]. The discussion is currently very general and superficial.

Answer: We sincerely thank you for your valuable suggestions. In accordance with your comments, we have comprehensively and systematically reorganized the Discussion section, and further clarified the similarities and differences between our study and the newly published literature. Please refer to the revised manuscript we have submitted.
