# Supplementary material for: Kisspeptin improves local ovarian insulin resistance in PCOS by modulating the PI3K/AKT/GLUT4 signaling pathway
Source: PLoS One. 2026 Feb 2;21(2):e0342158. doi: 10.1371/journal.pone.0342158 (PMC12863573; doi:10.1371/journal.pone.0342158)
Supplement: Attachment — Submitted filename: reviewer comments ffinal.docx [file pone.0342158.s002.docx]

The paper investigates the interaction between kisspeptin and insulin sensitivity in the ovaries, focusing on a PCOS mouse model. The study presents the concept and demonstrates that kisspeptin expression is altered in the ovaries of PCOS mice. Furthermore, external supplementation with kisspeptin enhanced GLUT4 expression, leading to improved glucose metabolism within the ovary. However the paper can be improved by doing the following:

1. **Introduction**:
   Please elaborate on how insulin sensitivity is maintained or enhanced in the ovaries despite systemic insulin resistance in PCOS. Discuss the key insulin signaling pathways involved (e.g., PI3K-Akt, MAPK) and their differential regulation in ovarian versus peripheral tissues.
   Additionally, the study reports downregulation of the Insulin- PI3K-Akt pathway in granulosa cells. However, some literature (e.g., PMID: 27060006) suggests insulin sensitivity in ovary under hyperandrogenic conditions with activation of insulin pathways. Please include this in the introduction.
2. Replace the term “kisspeptin antagonist” with “KISS1 receptor antagonist” or “KISS1R antagonist” throughout the manuscript for scientific accuracy.
3. In the **Methods** section, provide complete details of the in vivo model:
   - Duration of DHEA treatment for follicle collection. References supporting the DHEA model. What was the rationale for using the DHEA model vs DHT?
   - Time points for testosterone measurement following DHEA treatment.
   - Was insulin supplemented to the culture media for the primary granulosa cells. Were experiments done with and without above.
   - Please specify in methods and legends if kisspeptin and kisspeptin antagonist was added to PCOS-IR cells ?
   - Catalog numbers for all reagents and kits used (e.g., testosterone and insulin kits, PMSG, goat serum)
4. In **Figure 1A**:
   - Label the antral follicles and cysts clearly.
   - Add data on the body weight of mice at or near the time of sacrifice.
5. In **Figure 2**, clarify whether the study rationale is based on local kisspeptin production in the ovary or on circulating levels. Since kisspeptin was added to the culture media, this likely simulates serum levels-please clarify in discussion. If Kisspeptin Receptor expression was measured by any means, please include it in the paper.
6. For **Figure 3**, specify the number of biological and technical replicates used. Include this information in the **Methods** section and figure legend. Also, though mentioned in the legend, it may be helpful to call the control group PCOS-IR, since Control is vehicle+ HFD group in figure 2. Add Data from Control-IR animals in supplementary if available, to see the independent effect of kisspeptin.
7. (Same as point 7) Ensure that replicate numbers are clearly indicated for all experiments in Figure 4.
8. In the **Discussion**, please comment on the observation that the rise in testosterone in the model group compared to controls is relatively modest, yet the difference in glucose tolerance test (GTT) results and HOMA-IR is quite significant. This may suggest aggravated insulin resistance in the presence of slightly increased testosterone. Also would be interesting to add some details on if insulin resistance preceeds morphological changes in the ovary.
